# Age-related gene and miRNA expression changes in airways of healthy individuals

**DOI:** 10.1038/s41598-019-39873-0

**Published:** 2019-03-06

**Authors:** J. Ong, R. R. Woldhuis, I. M. Boudewijn, A. van den Berg, J. Kluiver, K. Kok, M. M. Terpstra, V. Guryev, M. de Vries, C. J. Vermeulen, W. Timens, M. van den Berge, C. A. Brandsma

**Affiliations:** 1 University of Groningen, University Medical Center Groningen, Department of Pathology and Medical Biology, Groningen, The Netherlands; 2 University of Groningen, University Medical Center Groningen, Groningen Research Institute for Asthma and COPD (GRIAC), Groningen, The Netherlands; 3 University of Groningen, University Medical Center Groningen, Department of Pulmonary Diseases, Groningen, The Netherlands; 4 University of Groningen, University Medical Center Groningen, Department of Genetics, Groningen, The Netherlands; 5 University of Groningen, University Medical Center Groningen, European Research Institute for the Biology of Ageing, Groningen, The Netherlands; 6 University of Groningen, University Medical Center Groningen, Department of Epidemiology, Groningen, The Netherlands

## Abstract

Knowledge on age-related miRNA changes in healthy individuals and their interaction with mRNAs is lacking. We studied age-related mRNA and miRNA expression changes and their interactions in normal airways. RNA and small RNA sequencing was performed on bronchial biopsies of 86 healthy individuals (age: 18–73) to determine age-related expression changes. Per age-related miRNA we determined the enrichment of age-related predicted targets and their correlation. We identified 285 age-related genes and 27 age-related miRNAs. Pathway enrichment showed that genes higher expressed with age were involved in synapse-related processes. Genes lower expressed with age were involved in cell cycle regulation, the immune system and DNA damage/repair. MiR-146a-5p, miR-146b-5p and miR-142-5p were lower expressed with increasing age and we found a significant enrichment for predicted targets of these miRNAs among genes that were higher expressed with age. The expression levels of the enriched predicted targets *RIMS2* and *IGSF1* were negatively correlated with both miR-146a-5p and miR-146b-5p. *RIMS2* was present in the enriched process, i.e. positive regulation of synaptic transmission. In conclusion, genes decreased with ageing are involved in several of the ageing hallmarks. Genes higher expressed with ageing were involved in synapse-related processes, of which *RIMS2* is potentially regulated by two age-related miRNAs.

## Introduction

Worldwide, the proportion of individuals over 60 years old is predicted to increase from 12% in 2015 to 22% in 2050^1^. This rise in the number of elderly individuals in the population will lead to an increase in ageing-associated diseases. Ageing is a process in which the body homeostasis progressively declines, resulting in increased risk of disease or death^2^. Nine hallmarks have been defined for ageing: genomic instability, telomere attrition, epigenetic alterations, loss of proteostasis, deregulated nutrient sensing, mitochondrial dysfunction, cellular senescence, stem cell exhaustion and altered intercellular communication^3^. In the ageing lung, dysregulation of the extracellular matrix has been proposed as an additional hallmark^4^.

During normal ageing, lung function declines over time due to a variety of mechanisms and anatomic changes including smaller thoracic cavity, reduced respiratory muscle function, senile emphysema and reduced mucus clearance^5^. Knowledge about changes in the airways due to ageing is scarce. Previously, it was shown that airway wall thickness was decreased with higher age^6^ and a murine study showed that senescence of airway progenitor cells impairs airway regeneration^7^.

It is likely that changes in gene and microRNA (miRNA) expression play a role in ageing-associated processes in the lung. To gain insight in these processes, several gene and miRNA expression studies have been performed. Previously, we identified 3,509 age-related genes in lung tissue that were involved in lung development, cell-cell contact, calcium signalling and immune response^8^. Dugo *et al*. found enrichment of genes involved in extracellular matrix production and function, pro-inflammatory responses and wound healing among the 217 age-related genes in lung tissue^9^. Multiple miRNAs have been proposed to be involved in the process of ageing^10–13^ and especially in cellular senescence, for example by targeting genes that play a role in the p53/p21 and p16/Rb senescence pathways^14^. In addition, expression levels of miR-210 and miR-494 were induced by DNA damage and oxidative stress in human foetal lung fibroblasts and vice versa. These miRNAs induced DNA damage and oxidative stress via a positive feedback loop^15^.

Although several genes and miRNAs have been suggested to be involved in ageing of the lungs, limited information is available about the underlying age-related mRNA-miRNA interactions. Moreover, so far no studies have been performed in healthy subjects with a normal pulmonary function and without respiratory complaints. In the present study, we aimed to identify age-related mRNA and miRNA changes and their interactions in bronchial biopsies of 86 healthy individuals with an age range of 18–73 years.

## Results

### Subject characteristics

After quality control, RNA sequencing data of 77 and small RNA sequencing data of 82 bronchial biopsies were available for further analyses, resulting in a total of 86 biopsies originating from subjects with an age range of 18–73 years (Table 1). Of these 86 subjects, 73 had both mRNA and small RNA sequencing data available.

**Table 1:** Subject characteristics.

Characteristics	Subjects
N	86
Male/Female, n	48/38
Age range, years	18–73
Never-smoker/smoker	44/42
FEV_1_, % predicted^a^	98.7 (93.6–107.7)
FEV_1_/FVC, %^b^	78.8 (74.5–84.1)
Pack-years, n	15.4 (4.4–29.3)

^a^FEV_1_, % predicted = percentage of Forced Expiratory Volume in one second of the predicted normal value for an individual of the same sex, age and height.

^b^FEV_1_/FVC, % = Forced Expiratory Volume in one second/Forced Vital Capacity ratio expressed in percentage.

Medians (interquartile ranges) are shown unless otherwise stated.

### Age-related genes in human bronchial biopsies

**Figure 1:**
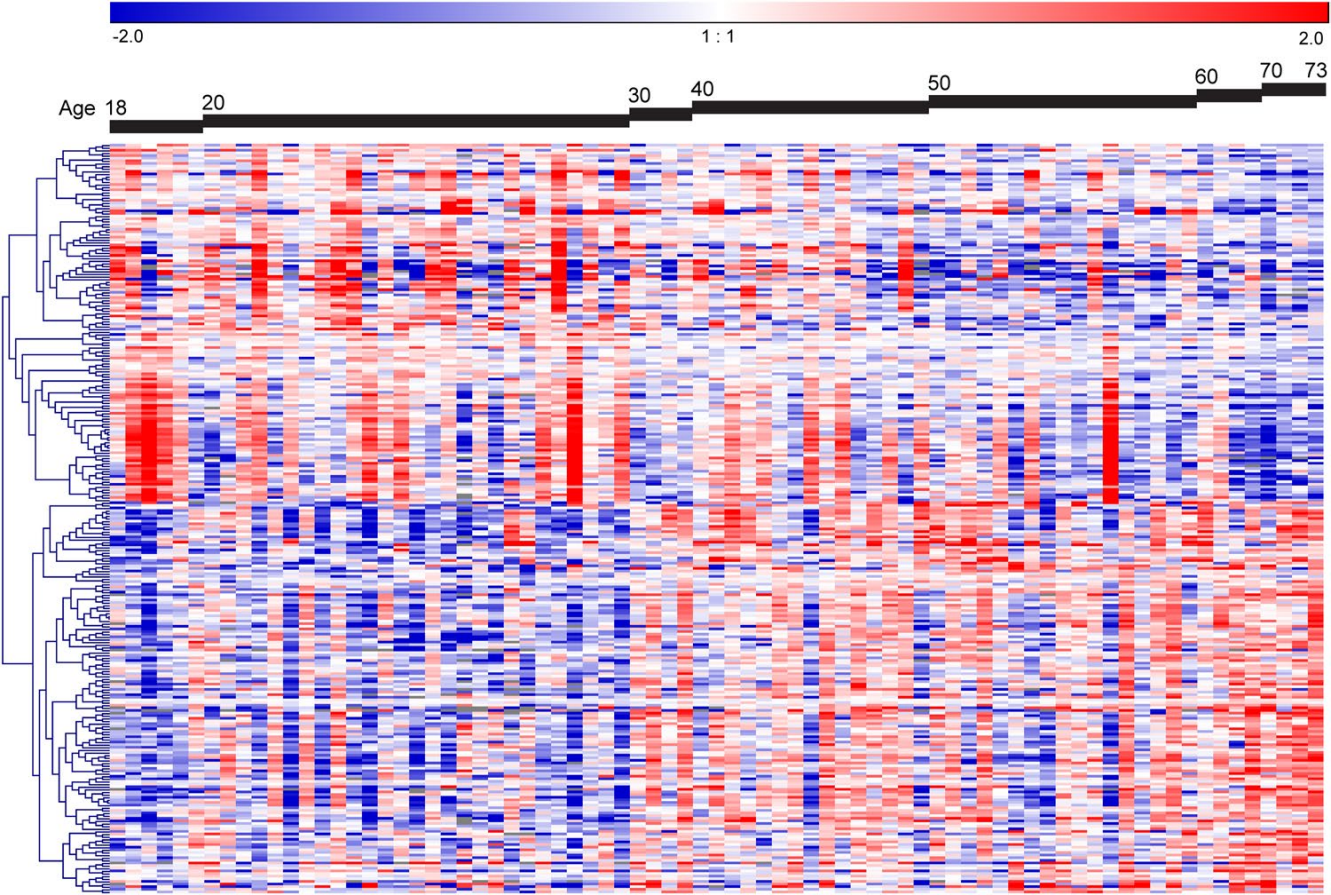
Age-related genes in human bronchial biopsies. Heatmap of the supervised hierarchical clustering of the 285 age-related genes. The 77 subjects were ordered by age. The heatmap shows the median-centered expression of the 285 genes of which 136 genes were lower expressed and 149 were higher expressed with increasing age (FDR adjusted p-value < 0.05).

Significant age-related changes in expression were observed for 285 genes (FDR adjusted p-value < 0.05, Fig. 1, Supplementary Table 1A,B). Of these genes, 149 were higher expressed and 136 were lower expressed with increasing age. The association with age of the top-3 higher expressed genes (*TMTC1*, *CPS1* and *RP11-550F7.1*) and top-3 lower expressed genes (*TIMELESS*, *KNTC1* and *BRIP1*) with lowest FDR adjusted p-values are shown in Supplementary Fig. 1A.

### Replication of age-related genes in lung tissue

Of the 149 higher expressed and the 136 lower expressed genes with increasing age, 117 and 118 genes, respectively, were also detected in our previously published lung tissue dataset^8^. In this dataset, we replicated the association with age for 58 out of 117 higher expressed genes (49.6%) and 43 out of 118 lower expressed genes (36.4%) (p-value < 0.05, Supplementary Table 1A,B).

### Biological processes and pathway enrichment of age-related genes

The biological processes and pathway enrichment analyses in Enrichr revealed amongst the age-related genes 155 and 116 significantly enriched processes and pathways respectively (FDR adjusted p-value < 0.05, Supplementary Table 2). The genes higher expressed with age were enriched for synapse-related processes (n = 19 genes) and pathway related to muscle contraction (n = 8 genes). The genes lower expressed with age were enriched for processes and pathways related to cell cycle (n = 54 genes), the immune system (n = 44 genes) and DNA damage and repair (n = 25 genes). Similar results were obtained using g:Profiler (Supplementary Table 3).

### Age-related miRNAs in human bronchial biopsies

**Figure 2:**
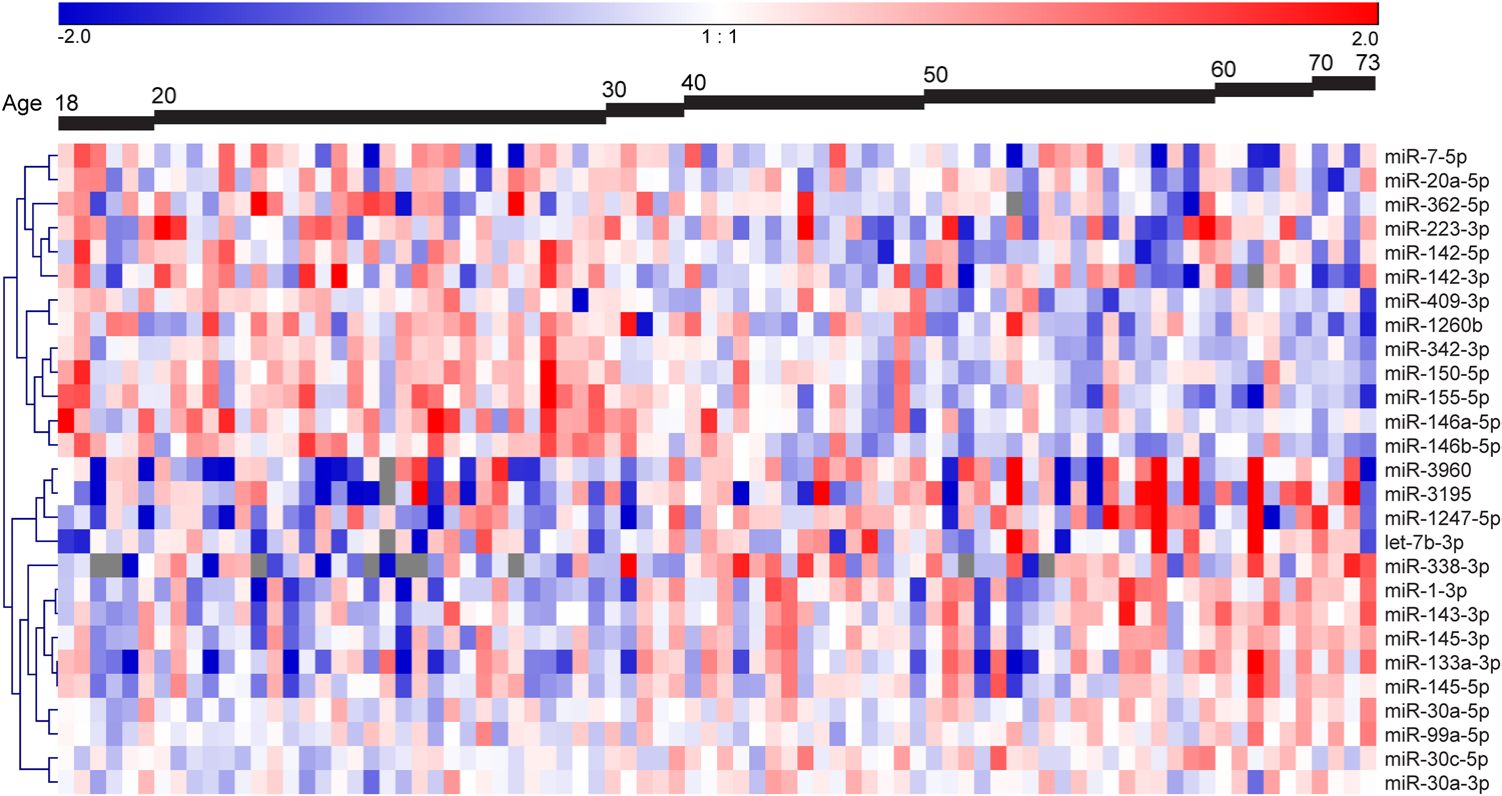
Age-related miRNAs in human bronchial biopsies. Heatmap of the supervised hierarchical clustering of the 27 age-related miRNAs. The 82 subjects were ordered by age. The heatmap shows the median-centered expression of the 27 miRNAs of which 14 miRNAs were higher expressed and 13 miRNAs were lower expressed with increasing age (FDR adjusted p-value < 0.05).

Significant age-related expression changes were observed for 27 miRNAs (FDR adjusted p-value < 0.05, Fig. 2, Supplementary Table 1C,D). This included 13 miRNAs with lower expression levels and 14 with higher expression levels with increasing age. The association with age for the top-3 higher expressed miRNAs (miR-3195, miR-1247-5p, and miR-1-3p) and top-3 lower expressed miRNAs (miR-146b-5p, miR-155-5p and miR-20a-5p) based on FDR adjusted p-values are shown in Supplementary Fig. 1B.

### Enrichment of age-related predicted miRNA target genes

**Figure 3:**
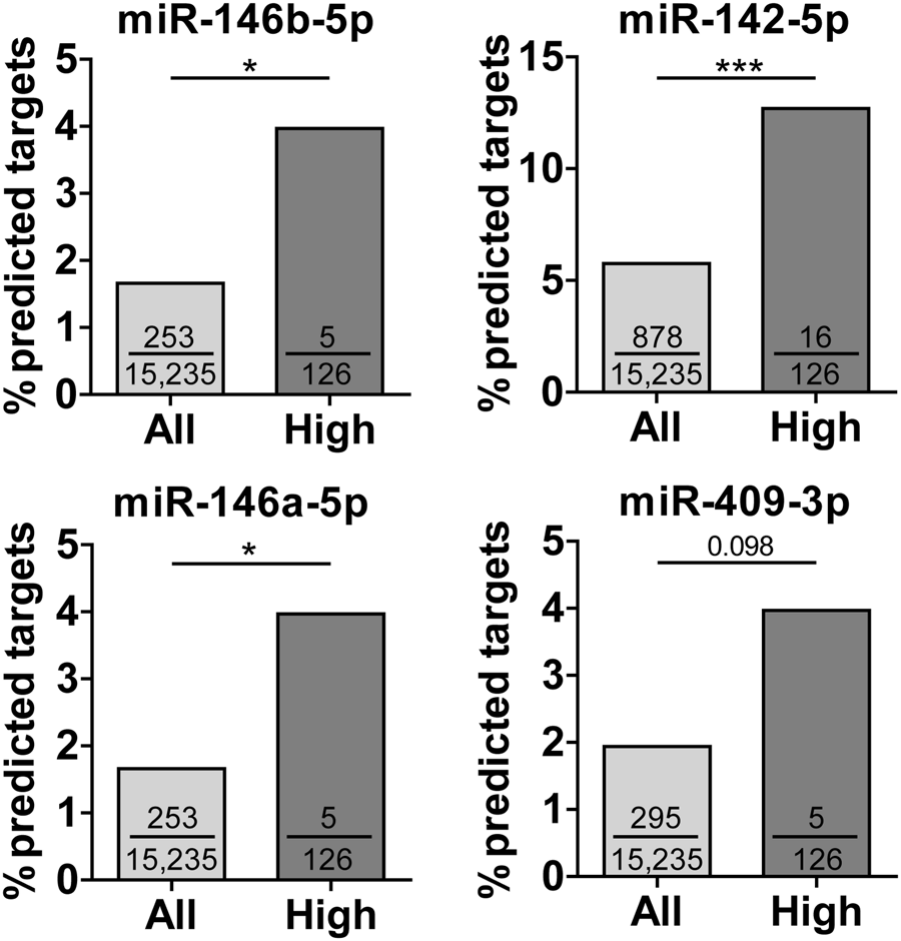
Enrichment of predicted miRNA target genes within the age-related genes. MiRNA target gene enrichment analysis for miRNAs that were lower expressed with increasing age. All = all expressed genes (n total = 15,235 genes); High = genes that were higher expressed with age (n = 126 genes); The numbers in the bars indicate the number of predicted target genes divided by all expressed genes (15,235 genes, light grey bars) or by the 126 genes that were higher expressed with age (dark grey bars); *p-value < 0.05, ***p-value < 0.001.

To identify potential interactions between age-related mRNA and miRNA expression changes, we determined whether the predicted target genes of age-related miRNAs were enriched among the genes that were either higher or lower expressed with age as compared to all expressed genes. Of the 13 miRNAs that were lower expressed with increasing age, miR-146b-5p, miR-142-5p and miR-146a-5p showed a significant enrichment of their predicted target genes among genes higher expressed with increasing age (p-value < 0.05), and a similar trend was observed for miR-409-3p (p-value = 0.098, Fig. 3). MiR-146b-5p, miR-142-5p and miR-146a-5p had five, 16 and five predicted targets that were higher expressed with increasing age, respectively (Supplementary Table 4). Of the 14 miRNAs that were higher expressed with increasing age, no significant enrichment of predicted target genes was found among genes lower expressed with increasing age.

### Correlation of miR-146b-5p, miR-142-5p and miR-146a-5p with their predicted targets

**Figure 4:**
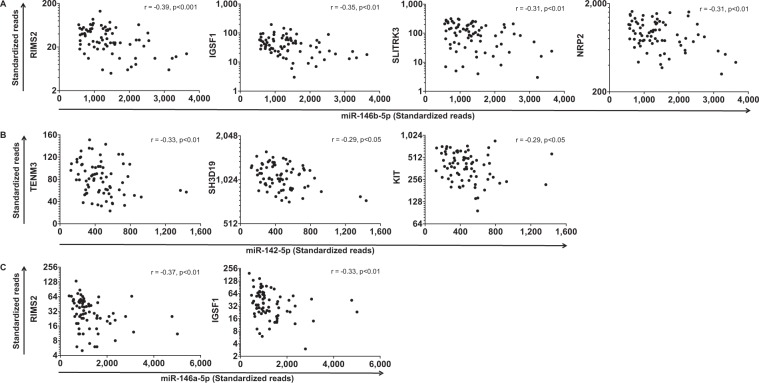
Correlation between miRNA expression and expression of their age-related predicted targets. Lower expressed miRNAs with increasing age, (**A**) miR-146b-5p, (**B**) miR-142-5p and (**C**) miR-146a-5p correlated with their predicted target genes that are higher expressed with age. Spearman’s correlation coefficient r and p-values are shown in the graphs.

To further substantiate the connection between age-related gene and miRNA expression changes, we assessed the correlation between miR-146b-5p, miR-142-5p and miR-146a-5p and their enriched predicted targets that were higher expressed with increasing age (Supplementary Table 4). We identified nine significant negative correlations between the three miRNAs and their age-related predicted target genes (Fig. 4). For miR-146b-5p, we found a significant negative correlation for four of the five enriched predicted target genes (Fig. 4A). For miR-142-5p, we found a negative correlation for three out of sixteen (Fig. 4B) and for miR-146a-5p for two out of five enriched predicted target genes (Fig. 4C). The nine significant negative correlations involved seven different target genes. Of these, *RIMS2* and *IGSF1* were negatively correlated with both miR-146b-5p and miR-146a-5p. *RIMS2* was the 4^th^ and *IGSF1* was the 6^th^ most significant gene with higher expression with age (Supplementary Table 1A).

## Discussion

In this study, we investigated the potential role of miRNAs in the ageing process in healthy airways by combining age-related miRNA and gene expression changes. We identified 285 genes and 27 miRNAs of which the expression levels were changed with increasing age in bronchial biopsies. The genes with higher expression levels with increasing age were mainly involved in synapse-related processes. The genes with lower expression levels with increasing age were mainly involved in DNA damage and repair, cell cycle regulation and the immune system. MiR-146b-5p, miR-142-5p and miR-146a-5p expression levels were lower with increasing age and a significant enrichment of their predicted target genes was found among the genes higher expressed with increasing age. *RIMS2* and *IGSF1* were negatively correlated with miR-146b-5p and miR-146a-5p. Of these predicted target genes, *RIMS2* was involved in positive regulation of synaptic transmission, one of the significantly enriched biological processes amongst the age-related genes. To our knowledge, this is the first study in which age-related genes were connected to age-related miRNAs in airway biopsies from respiratory healthy subjects.

Interestingly, the above-mentioned miRNAs have been associated with age in previous studies. In accordance with our study, the levels of miR-142-5p in human serum were lower with increasing age^16^. Different to our findings, the expression levels of miR-146a-5p were shown to be higher with increasing age in human mesenchymal stem cells^17^ and both miR-146a-5p and miR-146b-5p levels were increased in senescent compared to quiescent as well as proliferating human foreskin fibroblasts^18^. These disparate findings might be related to differences in cell type and/or tissue specific expression changes of these miRNAs with age. The host gene of miR-146a, i.e. *MIR3142HG*^19^, was also significantly lower expressed with age in our study (p-value < 0.05), which is in agreement with our findings for miR-146a.

Our study showed that genes with higher expression levels with increasing age in bronchial biopsies were involved in synapse-related processes. The nervous system of the respiratory tract regulates the calibre of the bronchi and pulmonary vessels, but how the nervous system of the respiratory tract changes during ageing is still poorly understood^20^. *RIMS2*, known to be involved in the synapse-related processes, is one of the genes with higher mRNA expression levels with age. More specifically, RIMS2 is a presynaptic protein in the synaptic vesicle release site, so called the active zone, and interacts with several synaptic proteins including Rab3 to regulate Ca^2+^-dependent neurotransmitter release from synaptic vesicles^21,22^. Previous study suggested that ageing may decrease the active zone density of neuromuscular junctions in mice and rats which may affect neurotransmission^23^. However, *RIMS2* is lowly expressed in our study and so far, no studies have shown an association between *RIMS2* and ageing human airways. We demonstrated that *RIMS2* expression was negatively correlated with two miRNAs that were lower expressed with age, i.e. miR-146b-5p and miR-146a-5p, suggesting that these miRNAs may regulate synapse-related changes during ageing.

In our correlation analyses, we found a significant negative correlation of *IGSF1* with expression levels of miR-146b-5p and miR-146a-5p, which are from the same miRNA seed family. This suggests that higher *IGSF1* expression is regulated by these miRNAs with increasing age. The *IGSF1* gene encodes an immunoglobulin superfamily glycoprotein of which the function and molecular mechanisms are not well defined yet^24^. Previous studies suggested that the IGSF1 protein may inhibit the TGF-β and activin signalling pathways by interacting with type I receptors^24,25^. As *IGSF1* is higher expressed with increasing age, the transcription of TGF-β- and activin-specific target genes might be negatively affected during ageing. The TGF-β pathway plays an important role in tissue repair and remodelling, and therefore the inhibition of this pathway may negatively affect the extracellular matrix protein production. Thus, our *IGSF1* findings would be in line with the previous observation of reduced airway wall thickness with ageing^6^. So far, the proposed correlations have not been experimentally validated^26^.

The genes with lower expression levels with increasing age were mainly involved in DNA damage and repair, cell cycle regulation and the immune system. These processes and pathways are linked to three of the hallmarks of ageing, i.e. genomic instability, cellular senescence and altered intercellular communication^4^. Furthermore, the top-3 genes with the most significantly decreased expression levels with increasing age, i.e. *TIMELESS*, *BRIP1* and *KNTC1*, were enriched in the processes/pathways DNA damage and repair, and/or cell cycle regulation. TIMELESS mediates DNA repair by binding to PARP1 that is involved in various DNA repair pathways and in maintaining genomic instability^27,28^. BRIP1 is a DNA helicase and has been reported to be involved in double-strand break repair by interacting with BRCA1^29^. In addition to DNA repair, the interaction between BRIP1 and BRCA1 is involved in DNA damage-induced checkpoint control during the G2 to M phase transition^30^. KNTC1 is a mitotic checkpoint regulator that checks whether the chromosomes are properly aligned during cell division^31^. The decreased expression levels of *TIMELESS*, *BRIP1* and *KNTC1* may thus contribute to the decline in DNA repair capacity during ageing.

For the replication of the age-related gene expression changes we used our previously published lung tissue dataset^8^. Despite differences in tissue origin and subject inclusion, we were able to replicate our findings for a substantial proportion of the genes, showing the robustness of our results. Several aspects might explain why not all genes could be replicated in the lung tissue dataset. For example, the cell composition and partly also the function of cells differ between bronchial biopsies derived from central airways and lung tissues derived from peripheral lung. Moreover, we only included bronchial biopsies from respiratory healthy subjects, while in the lung tissue dataset both patients with lung diseases and control subjects with normal lung function were included. One of the unique features of our study is that we used bronchial biopsies from respiratory healthy individuals (volunteers). This also brings us to the most important limitations, i.e. cross-sectional data, and availability of similar publicly available datasets for replication. As an alternative way of replication, we assessed the expression of six age-related miRNAs in lung tissue samples from 35 subjects with normal lung function with an age range of 42–82 years. We could not replicate the association with age for these miRNAs, as expression levels were quite variable in these samples and therefore power was limited (data not shown). Although our study showed negative correlations between the predicted and age-related target genes and the miRNAs within the same biopsies, additional experiments are required to show that these interactions can occur in relevant cell types present in the airways.

In conclusion, we identified changes in the expression of several genes and miRNAs and several potential mRNA-miRNA interactions in the airways during normal ageing. The genes that were lower expressed with increasing age are part of processes and pathways involved in three main hallmarks of ageing, i.e. genomic instability, cellular senescence and altered intercellular communication. The genes that were higher expressed with increasing age are involved in synapse-related processes, thus possibly in innervation of the airways of which *RIMS2* is potentially regulated by two age-related miRNAs miR-146b-5p and miR-146a-5p.

## Materials and Methods

### Bronchial biopsies from respiratory healthy subjects

We included bronchial biopsies (airway tissue samples) obtained from 94 respiratory healthy subjects participating in the study to obtain normal values of inflammatory variables from healthy smoking and never-smoking subjects (NORM study) (ClinicalTrials Identifier = NCT00848406^32^). Subjects did not have a history of lung disease, had no respiratory symptoms and had a normal lung function, which was defined by absence of bronchial hyperresponsiveness to methacholine (provocative concentration inducing a 20% fall in FEV_1_ (PC_20_) > 16 mg/ml), forced expiratory volume in one second/forced vital capacity (FEV_1_/FVC) higher than lower limit of normal and reversibility of the FEV_1_ to salbutamol <10%. The study was approved by the Medical Ethical Committee of the University Medical Center Groningen (METc 2009/007) and conducted according to the Good Clinical Practice guidelines. All subjects gave their written informed consent^32^.

### RNA and small RNA sequencing

RNA and small RNA sequencing was performed to assess changes in mRNA and miRNA expression, respectively. Total RNA was isolated from the bronchial biopsies using AllPrep DNA/RNA/miRNA Mini kit (Qiagen, Venlo, The Netherlands). RNA yield and quality was determined using the LabChip GX (Perkin Elmer, Waltham, MA, USA). The Ribo-Zero Gold kit (Illumina) was used to remove ribosomal RNA. RNA sequencing libraries were prepared using the TruSeq Stranded Total RNA Sample Preparation kit (Illumina) on the Caliper Sciclone NGS Workstation (PerkinElmer, Waltham, MA). Libraries were paired-end sequenced (2 × 100 bp) on the HiSeq 2500 (Illumina), which resulted in total read counts that passed the Illumina’s filter ranging from 44,780,324 to 113,186,108. FastQC (version 0.11.3) was used for the quality control of raw RNA sequencing data. HISAT (version 0.1.5^33^) and SAMtools (version 1.2^34^) were used for alignment to build b37 of the human reference genome and for sorting the aligned reads, respectively. Ensembl (release 75^35^) was used as the gene annotation database and the aligned reads were quantified with HTSeq (version 0.6.1p1). Quality control of the aligned reads was performed using Picard-tools (version 1.130). After filtering against genes with less than half of the samples achieving ≥5 standardized reads, 22,744 expressed genes were available for analyses. Small RNA libraries were generated with the NEXTflex Small RNA-seq kit V3 (Bioo Scientific, Uden, The Netherlands) without gel-based purification. The libraries were single-end sequenced (1 × 50 bp) on the Illumina HiSeq 2500 (Illumina, San Diego, CA, USA). Quality control was performed using FastQC (version 0.11.5). Trimming of the adapter sequences of the raw reads was performed using TrimGAlore 0.3.7. The miRDeep2 V2.0.0.8 software package^36^ was used to allocate the reads to the known human miRNAs (miRBase Release 21, http://www.mirbase.org/) allowing one mismatch. After filtering out miRNAs with a median expression <1 FPM, 518 miRNAs were available for analyses.

Of the available 94 bronchial biopsies, some samples were excluded from the analysis due to absence of RNA, poor alignment metrics, low total read counts (<100,000 reads) or technical errors during library preparation. In the small RNA sequencing data, principal component analysis showed an effect of library preparation batch on expression levels of the samples. Therefore these data were corrected for this factor in the subsequent analysis.

### Statistical analyses of (small) RNA sequencing data

The RNA and small RNA sequencing data analyses were performed using R version 3.3.2 with the Bioconductor-DESeq2 package (version 1.14.1). Associations between mRNA and miRNA expression and age were analysed using a generalized linear model. For these analyses, the following R-codes were run;

1. Design.mRNA = ~gender + current smoking + age
2. Design.miRNA = ~gender + batch + current smoking + age

Results were corrected for multiple testing using the Benjamini-Hochberg false discovery rate (FDR). Genes and miRNAs with an FDR adjusted p-value < 0.05 were considered statistically significant associated with age. Genesis software version 1.7.6 (Graz University of Technology, Graz, Austria)^37^ was used for supervised hierarchical clustering of the age-related mRNAs and miRNAs using Pearson’s correlation and to generate heatmaps. The subjects were ordered from young to old.

### Replication of age-related genes in lung tissues

To replicate the age-related gene expression changes, we compared the 285 age-related genes of our present study with our previously published lung tissue dataset^8^. In our previously published study, lung tissues obtained from 1,197 subjects (age range: 4–85 years) were used for the gene-expression signature for lung ageing. In total, 3,509 significantly differentially expressed genes with age were identified in lung tissue (FDR < 0.0001). In our present study, we compared the 285 age-related genes identified in bronchial biopsies with the 3,509 age-related genes identified in lung tissues. An age-related gene in our study was considered as replicated when the gene was also significantly related to age in the same direction in lung tissues at a nominal p-value < 0.05.

### Gene ontology and pathway analyses

Genes with higher and lower expression levels with age were separately subjected to Enrichr^38,39^ and g:Profiler^40^ to identify enriched biological processes (gene-set library: GO_Biological_Process_2018) and Reactome pathways (gene-set library: Reactome_2016 in Enrichr and Reactome_2018 in g:Profiler).

### Predicted target gene enrichment

The potential role of the miRNAs with age-related expression changes were explored by identifying enrichment of their predicted targets in the set of genes with higher and lower expression levels with age. For the analyses of enrichment of the age-related genes with predicted binding sites for the age-related miRNAs, we included the genes in RNA sequencing data that are also present in the gene list of TargetScan (version 7.1^41^). Of the 149 genes that are higher expressed with age and the 136 lower expressed genes, 126 genes and 127 genes, respectively, were present in TargetScan. Of all expressed genes in RNA sequencing data, 15,235 genes were present in TargetScan. Significant enrichment was assessed as percentages of predicted target genes in age-related genes with either higher (n = 126 genes) or lower (n = 127 genes) expression levels, respectively, as compared to percentages of predicted target genes among all expressed genes (n = 15,235). Differences were determined by Chi-square, considering a p-value smaller than 0.05 as significant. Correlation between gene and miRNA expression levels was assessed using the Spearman’s correlation (two-tailed). A correlation with p < 0.05 was considered statistically significant.

## Supplementary information

Supplementary Info

## Data Availability

The RNA sequencing and small RNA sequencing datasets used in this manuscript are available for collaboration upon request.
