# Supplementary material for: Age-related gene and miRNA expression changes in airways of healthy individuals
Source: Sci Rep. 2019 Mar 6;9:3765. doi: 10.1038/s41598-019-39873-0 (PMC6403379; doi:10.1038/s41598-019-39873-0)
Supplement: Supplementary file 1 — Supplementary Info [file 41598_2019_39873_MOESM1_ESM.pdf]

Age-related gene and miRNA expression changes in airways of healthy individuals

J Ong[1,2,#], RR Woldhuis[1,2,#], IM Boudewijn[2,3], A van den Berg[1], J Kluiver[1], K Kok[4], MM Terpstra[4], V Guryev[2,5], M de Vries[2,6], CJ Vermeulen[2,3], W Timens[1,2], M van den Berge[2,3,‡], CA Brandsma[1,2,‡,*]

[1]University of Groningen, University Medical Center Groningen, Department of Pathology and Medical Biology, Groningen, The Netherlands
[2]University of Groningen, University Medical Center Groningen, Groningen Research Institute for Asthma and COPD (GRIAC), Groningen, The Netherlands
[3]University of Groningen, University Medical Center Groningen, Department of Pulmonary Diseases, Groningen, The Netherlands
[4]University of Groningen, University Medical Center Groningen, Department of Genetics, Groningen, The Netherlands
[5]University of Groningen, University Medical Center Groningen, European Research Institute for the Biology of Ageing, Groningen, The Netherlands
[6]University of Groningen, University Medical Center Groningen, Department of Epidemiology, Groningen, The Netherlands

[#]Co-first authors
[‡]Co-last authors
[*]Corresponding author: CA Brandsma, E-mail: c.a.brandsma@umcg.nl

Contents

Supplementary Figure 1. Top-3 higher and lower expressed genes and miRNAs with increasing age in bronchial biopsies 2

Supplementary Table 1. Results of the age-related genes and miRNAs obtained from the generalized linear model 3

Table 1A. Genes higher expressed with age 3

Table 1B. Genes lower expressed with age 6

Table 1C. MiRNAs higher expressed with age 8

Table 1D. MiRNAs lower expressed with age 8

Supplementary Table 2. Gene ontology and pathway analyses using Enrichr 9

Table 2A. Biological processes of the genes higher expressed with age 10

Table 2B. Reactome pathways of the genes higher expressed with age 11

Table 2C. Biological processes of the genes lower expressed with age 12

Table 2D. Reactome pathways of the genes lower expressed with age 21

Supplementary Table 3. Gene ontology and pathway analyses using g:Profiler 30

Table 3A. Biological processes of the genes higher expressed with age 31

Table 3B. Reactome pathways of the genes higher expressed with age 32

Table 3C. Biological processes of the genes lower expressed with age 33

Table 3D. Reactome pathways of the genes lower expressed with age 36

Supplementary Table 4. Results of the age-related predicted target gene enrichment analyses 38

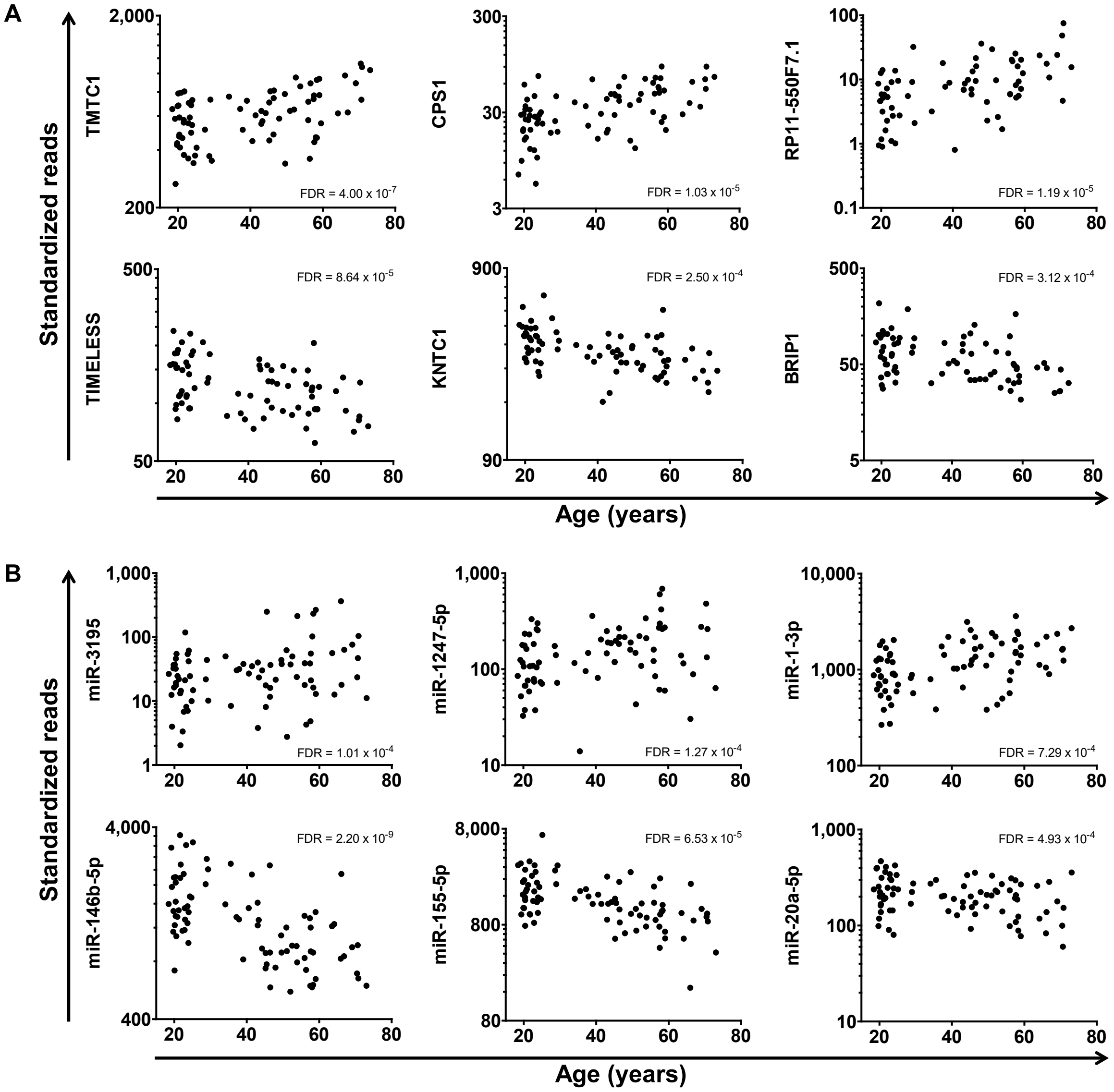

Supplementary Figure 1. Top-3 higher and lower expressed genes and miRNAs with increasing age in bronchial biopsies. The correlation plots with age of the (A) genes and (B) miRNAs that had the lowest FDR adjusted p-values were made using unadjusted data. FDR adjusted p-values are shown in the graphs.

Supplementary Table 1. Results of the age-related genes and miRNAs obtained from the generalized linear model

Table 1A. Genes higher expressed with age

Genes		Generalized linear model		Lung tissue	
Higher expressed with age	Mean expression	FC/year	FDR	Expressed	Replicated
TMTC1	627	1.011	4.00E-07	Yes	Yes
CPS1	37	1.019	1.03E-05	Yes	Yes
RP11-550F7.1	10	1.034	1.19E-05		
RIMS2	35	1.021	2.97E-05	Yes	
AP000688.8	48	1.023	5.65E-05		
IGSF1	46	1.021	1.24E-04	Yes	
CCL11	31	1.030	1.49E-04	Yes	Yes
PRIMA1	34	1.025	4.23E-04	Yes	Yes
PLEKHH2	621	1.012	4.33E-04	Yes	Yes
GPX3	532	1.015	7.67E-04	Yes	Yes
HDC	73	1.018	1.06E-03	Yes	Yes
PCDH11X	11	1.041	1.43E-03		
PCDHB9	20	1.018	1.52E-03	Yes	
SLC8A3	12	1.022	1.55E-03	Yes	Yes
PDZRN4	19	1.025	1.58E-03	Yes	Yes
CTD-2269F5.1	24	1.014	1.60E-03		
PCSK2	31	1.023	2.01E-03	Yes	
PCDH9	70	1.015	2.10E-03	Yes	
EDA2R	38	1.011	2.18E-03	Yes	Yes
LRP1B	197	1.019	2.26E-03	Yes	
SYT8	60	1.015	2.26E-03	Yes	Yes
NAALAD2	66	1.011	2.44E-03	Yes	Yes
TRIM9	31	1.017	2.51E-03	Yes	Yes
TMOD2	296	1.009	2.53E-03	Yes	
GADL1	38	1.025	2.53E-03	Yes	
RIMS4	19	1.018	2.53E-03	Yes	
FGF9	32	1.015	2.78E-03	Yes	
NR3C2	474	1.005	2.91E-03	Yes	
NELL1	18	1.021	2.91E-03	Yes	
RP11-15K2.2	13	1.024	2.97E-03		
KLHL23	154	1.011	3.04E-03	Yes	
EDIL3	598	1.013	3.71E-03	Yes	
AC004538.3	26	1.015	4.05E-03		
TNNI2	13	1.019	4.29E-03	Yes	Yes
LINC00691	12	1.019	4.36E-03		
LPHN3	81	1.016	4.50E-03	Yes	Yes
CTC-260E6.6	30	1.020	4.50E-03		
UGT1A10	21	1.021	4.50E-03		
NRXN1	138	1.015	5.76E-03	Yes	
PCDHB13	40	1.011	6.43E-03	Yes	
HPGD	262	1.016	6.72E-03	Yes	
KCNIP2	41	1.014	6.72E-03	Yes	
TMEM252	13	1.018	6.72E-03		
ASPA	63	1.014	6.92E-03	Yes	Yes
PCDHB4	31	1.012	7.83E-03	Yes	Yes
DIO3	11	1.025	8.00E-03	Yes	
DDIT4L	43	1.017	9.62E-03	Yes	Yes
NEGR1	302	1.014	1.16E-02	Yes	Yes
AMIGO2	178	1.007	1.26E-02	Yes	Yes
CDH19	96	1.020	1.26E-02	Yes	
DGKB	35	1.020	1.26E-02	Yes	
TRHDE-AS1	22	1.019	1.26E-02		
DIO3OS	90	1.018	1.28E-02	Yes	
L1CAM	82	1.020	1.31E-02	Yes	Yes
RP1-142L7.5	29	1.017	1.32E-02		

Table 1A (Continued)

Genes		Generalized linear model		Lung tissue	
Higher expressed with age	Mean expression	FC/year	FDR	Expressed	Replicated
PCDHB12	23	1.015	1.33E-02	Yes	Yes
KIT	414	1.011	1.49E-02	Yes	
PRDM5	182	1.008	1.51E-02	Yes	
MPP6	400	1.007	1.51E-02	Yes	
FMO3	373	1.011	1.51E-02	Yes	Yes
NCAM1	63	1.013	1.61E-02	Yes	
PRKCA	483	1.004	1.62E-02	Yes	
ZNF676	16	1.018	1.62E-02	Yes	Yes
PCDHB14	58	1.009	1.67E-02	Yes	Yes
XKR4	106	1.019	1.70E-02	Yes	
RP11-468H14.2	15	1.012	1.76E-02		
RP11-332H18.4	25	1.014	1.78E-02		
GRIK3	51	1.019	1.86E-02	Yes	
MTUS2	18	1.023	1.99E-02		
SBDSP1	165	1.006	2.13E-02		
AC007362.3	30	1.012	2.13E-02		
SLITRK3	119	1.024	2.17E-02	Yes	Yes
ZKSCAN8	567	1.003	2.17E-02		
TENM3	81	1.009	2.17E-02		
C9orf47	31	1.018	2.17E-02	Yes	Yes
SLC12A1	13	1.020	2.17E-02	Yes	
MME	215	1.017	2.26E-02	Yes	
RP3-525N10.2	15	1.017	2.30E-02		
NCAM2	62	1.016	2.32E-02	Yes	Yes
EPHA7	284	1.012	2.37E-02	Yes	
ADAMTS6	68	1.012	2.42E-02	Yes	
AJAP1	146	1.015	2.49E-02	Yes	Yes
PCDHB10	21	1.014	2.49E-02	Yes	Yes
NRP2	939	1.009	2.59E-02	Yes	
ANGPT1	179	1.012	2.59E-02	Yes	Yes
VGLL3	322	1.010	2.60E-02	Yes	Yes
PCDHB6	10	1.015	2.82E-02	Yes	Yes
CACNB2	144	1.009	2.83E-02	Yes	
FAM13C	174	1.009	2.83E-02		
WNT2B	287	1.006	2.83E-02	Yes	
C6	89	1.029	2.83E-02	Yes	Yes
TBX5	81	1.015	2.83E-02	Yes	Yes
CNKSR2	61	1.012	2.83E-02	Yes	Yes
PMP2	31	1.024	2.83E-02	Yes	
FGF14-AS2	30	1.009	2.83E-02		
TPSD1	10	1.027	2.87E-02	Yes	
DIRAS3	31	1.012	2.88E-02	Yes	Yes
PCDHGA5	43	1.010	2.91E-02	Yes	
FIBIN	76	1.013	2.93E-02		
RAB44	15	1.014	3.00E-02		
TMEFF2	28	1.013	3.03E-02	Yes	Yes
LOC100421166	50	1.013	3.07E-02		
AK5	22	1.012	3.07E-02	Yes	
SLC24A2	18	1.028	3.07E-02	Yes	
TBX4	162	1.015	3.08E-02	Yes	Yes
CDR1	136	1.021	3.08E-02	Yes	Yes
RP11-268J15.5	21	1.013	3.08E-02		
HAP1	42	1.014	3.36E-02	Yes	
HPSE2	91	1.017	3.46E-02	Yes	Yes
GAS2	34	1.010	3.46E-02	Yes	
PHYH	123	1.006	3.51E-02	Yes	
CACNA2D3	70	1.009	3.51E-02	Yes	Yes
FAT3	281	1.012	3.53E-02	Yes	
PGR	157	1.014	3.54E-02	Yes	Yes
FBXO32	707	1.006	3.59E-02	Yes	Yes

Table 1A (Continued)

Genes		Generalized linear model		Lung tissue	
Higher expressed with age	Mean expression	FC/year	FDR	Expressed	Replicated
LIN7A	58	1.010	3.59E-02	Yes	
GHR	138	1.006	3.62E-02	Yes	Yes
PRKG2	37	1.010	3.62E-02	Yes	
DLG2	117	1.006	3.70E-02	Yes	Yes
RASD2	84	1.015	3.82E-02	Yes	Yes
NTS	802	1.020	3.85E-02	Yes	
FGF14-IT1	64	1.011	3.85E-02		
SORCS1	55	1.012	3.91E-02	Yes	
TMOD1	48	1.015	3.97E-02	Yes	Yes
KCNMB4	26	1.012	3.97E-02	Yes	
HOXA2	16	1.015	3.97E-02	Yes	Yes
ZNF438	115	1.005	3.98E-02	Yes	
MYOC	68	1.024	3.98E-02	Yes	Yes
ROBO2	351	1.011	4.08E-02	Yes	Yes
SLC23A2	843	1.005	4.08E-02	Yes	Yes
CCBE1	76	1.014	4.08E-02	Yes	
MPPED2	75	1.008	4.08E-02	Yes	Yes
UCHL1	70	1.020	4.28E-02	Yes	
SNX15	24	1.010	4.41E-02	Yes	
CSMD3	44	1.030	4.50E-02	Yes	Yes
RP11-88I18.2	38	1.016	4.59E-02		
MLTK	698	1.009	4.71E-02		
SERPINB11	608	1.013	4.76E-02	Yes	
NTPCR	360	1.004	4.80E-02		
SH3D19	1,082	1.004	4.80E-02	Yes	
PLAT	186	1.011	4.80E-02	Yes	Yes
GPM6A	53	1.017	4.86E-02	Yes	
HOXA-AS2	40	1.014	4.86E-02		
RASL11B	23	1.016	4.86E-02	Yes	Yes
PDE5A	1,588	1.012	4.88E-02	Yes	Yes
SLC22A15	110	1.006	4.88E-02	Yes	
PCDHGA4	33	1.010	4.88E-02	Yes	
TUSC7	10	1.021	5.00E-02		
HSPA12A	94	1.011	5.00E-02	Yes	Yes

Table 1B. Genes lower expressed with age

Genes		Generalized linear model		Lung tissue	
Lower expressed with age	Mean expression	FC/year	FDR	Expressed	Replicated
TIMELESS	128	-1.010	8.64E-05	Yes	
KNTC1	341	-1.008	2.50E-04	Yes	
BRIP1	63	-1.016	3.12E-04	Yes	
E2F1	17	-1.019	4.33E-04	Yes	Yes
BLNK	103	-1.009	1.52E-03	Yes	
BLM	47	-1.020	1.53E-03	Yes	
ASPM	115	-1.023	1.58E-03	Yes	Yes
IQGAP3	48	-1.023	1.58E-03	Yes	Yes
MKI67	249	-1.022	1.71E-03	Yes	Yes
CPXM1	26	-1.015	2.05E-03	Yes	
BRCA2	142	-1.011	2.18E-03	Yes	Yes
DTL	34	-1.020	2.26E-03	Yes	Yes
CENPF	224	-1.016	2.51E-03	Yes	Yes
NUP210	369	-1.010	2.59E-03	Yes	
NCAPH	26	-1.018	2.86E-03	Yes	
NOX5	20	-1.023	2.95E-03	Yes	
MCM5	247	-1.007	3.52E-03	Yes	
PSD4	734	-1.005	4.29E-03	Yes	
TACC3	65	-1.011	4.29E-03	Yes	
XKRX	114	-1.009	6.26E-03	Yes	Yes
CASC5	87	-1.016	7.83E-03	Yes	Yes
SLC13A2	42	-1.024	7.83E-03	Yes	Yes
NUSAP1	62	-1.019	8.00E-03	Yes	Yes
LMNB1	112	-1.013	8.53E-03	Yes	Yes
INSR	1,868	-1.006	9.03E-03	Yes	
ARHGAP26	684	-1.006	1.02E-02	Yes	
BTN2A2	146	-1.008	1.05E-02	Yes	
CDCA2	21	-1.021	1.10E-02	Yes	
TOP2A	242	-1.019	1.11E-02	Yes	Yes
FANCD2	111	-1.008	1.13E-02	Yes	
DIAPH3	20	-1.021	1.17E-02	Yes	
KIF14	26	-1.019	1.19E-02	Yes	Yes
HSH2D	119	-1.010	1.21E-02	Yes	
MYO3A	27	-1.021	1.25E-02	Yes	
ITGAE	193	-1.012	1.26E-02	Yes	Yes
LIMD1	519	-1.004	1.32E-02		
HCK	62	-1.014	1.33E-02	Yes	
RFWD3	272	-1.005	1.44E-02	Yes	
WHSC1	705	-1.005	1.45E-02	Yes	Yes
MERTK	179	-1.006	1.49E-02	Yes	
HAPLN3	69	-1.011	1.51E-02	Yes	
CENPE	66	-1.016	1.51E-02	Yes	Yes
IGHV4-34	18	-1.031	1.51E-02		
IGHD	443	-1.037	1.62E-02	Yes	Yes
HIST1H2AJ	31	-1.024	1.62E-02	Yes	Yes
ASF1B	27	-1.017	1.67E-02	Yes	
PIK3AP1	123	-1.015	2.08E-02	Yes	Yes
IDO1	78	-1.036	2.17E-02		
CXCL10	44	-1.033	2.26E-02	Yes	
TAP1	370	-1.013	2.27E-02	Yes	
HLA-B	3,505	-1.014	2.30E-02	Yes	
FANCA	74	-1.009	2.42E-02	Yes	
BUB1B	37	-1.014	2.42E-02	Yes	Yes
CDC20	35	-1.017	2.46E-02	Yes	
TNFRSF11A	74	-1.014	2.49E-02	Yes	
HMGA2	92	-1.009	2.57E-02	Yes	
LAMP3	105	-1.013	2.59E-02	Yes	Yes
ARHGAP11A	66	-1.012	2.59E-02	Yes	Yes
UBE2C	15	-1.024	2.59E-02	Yes	Yes

Table 1B (Continued)

Genes		Generalized linear model		Lung tissue	
Lower expressed with age	Mean expression	FC/year	FDR	Expressed	Replicated
PARP14	2,079	-1.007	2.83E-02	Yes	
HIVEP1	633	-1.004	2.83E-02	Yes	Yes
CENPU	56	-1.010	2.83E-02		
KIF15	33	-1.015	2.83E-02	Yes	Yes
MYBL2	16	-1.022	2.83E-02	Yes	
IGKV3-15	15	-1.029	2.83E-02		
CD52	34	-1.017	2.86E-02	Yes	
SSH2	470	-1.004	2.87E-02	Yes	Yes
IGKV3-11	42	-1.026	2.87E-02		
RP11-1220K2.2	48	-1.042	2.89E-02		
ZDHHC7	557	-1.003	2.90E-02	Yes	
MCM10	15	-1.023	2.90E-02	Yes	
ZNF518B	430	-1.005	2.91E-02	Yes	Yes
RHBDF2	177	-1.008	2.91E-02	Yes	
HLA-DMA	261	-1.011	2.91E-02	Yes	
C1orf51	65	-1.015	2.91E-02	Yes	
ZNF367	32	-1.013	3.03E-02	Yes	Yes
EPHX3	24	-1.017	3.03E-02		
NLRC5	893	-1.011	3.05E-02	Yes	
MAP7D2	14	-1.015	3.05E-02	Yes	
AURKB	13	-1.020	3.05E-02	Yes	
ABCC4	646	-1.005	3.07E-02	Yes	Yes
KIF11	113	-1.011	3.07E-02	Yes	Yes
RAB42	10	-1.014	3.07E-02		
ATAD2	327	-1.006	3.30E-02	Yes	
IL2RB	224	-1.010	3.34E-02	Yes	
HJURP	20	-1.017	3.42E-02	Yes	Yes
ERBB3	1,218	-1.006	3.46E-02	Yes	Yes
KIFC1	38	-1.015	3.51E-02	Yes	Yes
SLC34A2	865	-1.012	3.54E-02		
DENND1C	125	-1.007	3.59E-02	Yes	Yes
CIT	268	-1.009	3.61E-02	Yes	Yes
RRM2	79	-1.019	3.61E-02	Yes	Yes
TPX2	51	-1.016	3.65E-02	Yes	
HIST1H1A	22	-1.017	3.69E-02	Yes	
MYO7A	53	-1.014	3.72E-02	Yes	
ESPL1	38	-1.016	3.72E-02	Yes	Yes
AIM2	21	-1.017	3.72E-02		
TRANK1	834	-1.005	3.72E-02		
OAS3	343	-1.007	3.89E-02	Yes	
LPO	158	-1.049	3.97E-02	Yes	Yes
KIF23	57	-1.013	3.97E-02	Yes	
COL22A1	18	-1.015	3.98E-02	Yes	Yes
SLAMF8	37	-1.012	4.02E-02	Yes	
NAPSB	53	-1.013	4.06E-02	Yes	
HLA-DOA	188	-1.015	4.08E-02	Yes	
CHEK1	46	-1.010	4.08E-02	Yes	Yes
IGLV3-25	23	-1.038	4.08E-02	Yes	
GZMB	18	-1.022	4.08E-02	Yes	
CD7	21	-1.016	4.21E-02	Yes	
BUB1	50	-1.016	4.29E-02	Yes	Yes
CD79A	44	-1.026	4.29E-02	Yes	
HELLS	327	-1.007	4.41E-02	Yes	
KEL	65	-1.011	4.43E-02	Yes	
TLR10	28	-1.024	4.46E-02	Yes	
ORC1	21	-1.014	4.46E-02		
HIST1H2BL	22	-1.018	4.47E-02	Yes	
CD97	307	-1.007	4.59E-02	Yes	
JAK3	186	-1.012	4.65E-02	Yes	
GINS1	42	-1.009	4.65E-02	Yes	

Table 1B (Continued)

Genes		Generalized linear model		Lung tissue	
Lower expressed with age	Mean expression	FC/year	FDR	Expressed	Replicated
IGHV3-21	30	-1.032	4.65E-02		
SPATA32	15	-1.011	4.65E-02		
HLA-F	334	-1.011	4.69E-02	Yes	
RMI2	28	-1.013	4.71E-02		
DTX3L	715	-1.007	4.81E-02	Yes	
MARCKSL1	135	-1.011	4.86E-02	Yes	
PTPN6	306	-1.005	4.86E-02	Yes	
EPB41	498	-1.004	4.86E-02	Yes	
SIRPG	10	-1.022	4.86E-02	Yes	
PSME4	1,226	-1.004	4.89E-02	Yes	Yes
GCN1L1	1,392	-1.003	4.93E-02	Yes	
BTN3A1	496	-1.009	4.93E-02	Yes	
LGALS9	147	-1.006	4.93E-02		
SLC26A4	36	-1.035	4.97E-02	Yes	
DHX16	401	-1.003	4.99E-02	Yes	
STAT1	1,754	-1.013	4.99E-02	Yes	
IGLV3-1	18	-1.028	5.00E-02		

Table 1C. MiRNAs higher expressed with age

miRNAs		Generalized linear model	
Higher expressed with age	Mean expression	FC/year	FDR
miR-3195	44	1.03	1.01E-04
miR-1247-5p	186	1.02	1.27E-04
miR-1-3p	1,301	1.015	7.29E-04
miR-3960	126	1.025	7.78E-04
miR-133a-3p	314	1.017	1.44E-03
miR-145-5p	5,495	1.011	2.23E-03
miR-145-3p	1,146	1.01	3.22E-03
let-7b-3p	77	1.015	1.39E-02
miR-143-3p	51,915	1.009	1.83E-02
miR-30a-5p	3,038	1.005	3.88E-02
miR-30c-5p	5,276	1.006	4.55E-02
miR-99a-5p	3,018	1.005	4.55E-02
miR-338-3p	16	1.019	4.55E-02
miR-30a-3p	613	1.005	4.98E-02

Table 1D. MiRNAs lower expressed with age

miRNAs		Generalized linear model	
Lower expressed with age	Mean expression	FC/year	FDR
miR-146b-5p	1,407	-1.018	2.20E-09
miR-155-5p	1,592	-1.016	6.53E-05
miR-20a-5p	216	-1.012	4.93E-04
miR-142-5p	480	-1.013	7.29E-04
miR-342-3p	1,149	-1.008	1.77E-03
miR-150-5p	2,338	-1.011	2.01E-03
miR-146a-5p	1,313	-1.012	4.45E-03
miR-409-3p	59	-1.009	1.27E-02
miR-142-3p	64	-1.013	1.63E-02
miR-362-5p	29	-1.012	1.71E-02
miR-1260b	548	-1.009	4.55E-02
miR-223-3p	1,375	-1.011	4.55E-02
miR-7-5p	63	-1.011	4.55E-02

Supplementary Table 2. Gene ontology and pathway analyses using Enrichr

Table 2A. Biological processes of the genes higher expressed with age

Term	Overlap	P-value	Adjusted P-value	Z-score	Combined Score	Genes
synapse assembly (GO:0007416)	9/64	1.08E-09	8.33E-07	-1.773	36.614	GPM6A;NRXN1;PCDHB6;PCDHB14;PCDHB13; PCDHB4;PCDHB12;PCDHB10;PCDHB9
nervous system development (GO:0007399)	18/456	8.96E-09	3.46E-06	-1.326	24.563	ROBO2;GPM6A;PCDHGA5;PCDHGA4;NRXN1; TMOD2;PCDHB14;PCDHB13;PCDHB12;L1CAM; PCDHB10;NELL1;DLG2;MPPED2; PCDHB6;PCDHB4;HAP1;PCDHB9
calcium-dependent cell-cell adhesion via plasma membrane cell adhesion molecules (GO:0016339)	7/42	2.40E-08	5.79E-06	-1.508	26.465	PCDHB6;PCDHB14;PCDHB13;PCDHB4; PCDHB10;PCDHB9;CDH19
chemical synaptic transmission (GO:0007268)	14/290	3.75E-08	5.79E-06	-1.349	23.061	KCNIP2;NRXN1;GRIK3;PCDHB14;PCDHB13; PCDHB12;PCDHB10;CACNB2;DLG2; PCDHB6;KCNMB4;PCDHB4;HAP1;PCDHB9
anterograde trans-synaptic signaling (GO:0098916)	13/241	3.21E-08	5.79E-06	-1.206	20.817	KCNIP2;NRXN1;PCDHB14;PCDHB13;PCDHB12; PCDHB10;CACNB2;DLG2;PCDHB6; KC-NMB4;PCDHB4;HAP1;PCDHB9
cell-cell adhesion via plasma-membrane adhesion molecules (GO:0098742)	10/143	1.19E-07	1.54E-05	-1.174	18.721	ROBO2;TENM3;AMIGO2;PCDHB6;PCDHB14; PCDHB13;PCDHB4;PCDHB10;PCDHB9;CDH19
positive regulation of synaptic transmission (GO:0050806)	6/68	1.16E-05	1.28E-03	-1.419	16.128	RIMS2;SLC24A2;SLC8A3;RIMS4;NRXN1;HAP1
homophilic cell adhesion via plasma membrane adhesion molecules (GO:0007156)	5/65	1.24E-04	1.20E-02	-1.399	12.585	ROBO2;TENM3;AMIGO2;PCDHB6;CDH19

Table 2B. Reactome pathways of the genes higher expressed with age

Term	Overlap	P-value	Adjusted P-value	Z-score	Combined Score	Genes
Muscle contraction_Homo sapiens_R-HSA-397014	8/196	1.13E-04	3.17E-02	-2.100	19.085	TMOD1;SLC8A3;CACNB2;KCNIP2;TMOD2;TBX5; CACNA2D3;TNNI2
cGMP effects_Homo sapiens_R-HSA-418457	3/18	3.05E-04	4.28E-02	-1.955	15.828	KCNMB4;PRKG2;PDE5A

Table 2C. Biological processes of the genes lower expressed with age

Term	Overlap	P-value	Adjusted P-value	Z-score	Combined Score	Genes
regulation of mitotic cell cycle phase transition (GO:1901990)	11/185	5.62E-08	2.99E-05	-1.919	32.031	CDC20;CENPE;TPX2;CENPF;UBE2C;PSME4; E2F1;KIF14;BUB1B;KNTC1;PTPN6
DNA metabolic process (GO:0006259)	14/315	3.28E-08	2.99E-05	-1.364	23.499	TOP2A;HELLS;BLM;RMI2;FANCA;MCM10; BRCA2;BRIP1;ORC1;FANCD2;CHEK1; PSME4;TIMELESS;MCM5
mitotic nuclear division (GO:0140014)	7/75	7.77E-07	9.19E-05	-2.222	31.254	TPX2;ESPL1;KIFC1;UBE2C;NUSAP1;MYBL2; KIF11
DNA repair (GO:0006281)	12/289	6.80E-07	9.19E-05	-1.706	24.220	BLM;BRIP1;RMI2;DTX3L;FANCD2;RFWD3; CHEK1;PSME4;FANCA;TIMELESS;HMGA2;BRCA2
microtubule cytoskeleton organization involved in mitosis (GO:1902850)	6/45	5.80E-07	9.19E-05	-1.645	23.622	CENPE;ESPL1;NUSAP1;TACC3;KIF11;AURKB
mitotic spindle assembly (GO:0090307)	6/47	7.56E-07	9.19E-05	-1.482	20.889	TPX2;KIFC1;KIF23;MYBL2;KIF11;AURKB
cellular response to DNA damage stimulus (GO:0006974)	13/330	4.16E-07	9.19E-05	-1.348	19.800	TOP2A;BLM;DTX3L;FANCA;MCM10;BRIP1;DTL; FANCD2;RFWD3;CHEK1;PSME4;E2F1;TIMELESS
mitotic spindle organization (GO:0007052)	7/75	7.77E-07	9.19E-05	-1.386	19.493	CENPE;TPX2;KIFC1;KIF23;MYBL2;KIF11; AURKB
cytoskeleton-dependent cytokinesis (GO:0061640)	7/71	5.33E-07	9.19E-05	-1.243	17.959	ESPL1;NUSAP1;KIF23;NOX5;BRCA2;AURKB; CIT
mitotic sister chromatid segregation (GO:0000070)	7/83	1.55E-06	1.65E-04	-1.364	18.243	CENPE;ESPL1;KIFC1;KIF14;NUSAP1;KIF23; NCAPH
DNA replication (GO:0006260)	8/121	1.75E-06	1.70E-04	-1.546	20.491	BLM;BRIP1;RMI2;ORC1;CHEK1;TIMELESS; MCM10;MCM5
centromere complex assembly (GO:0034508)	5/37	4.94E-06	4.38E-04	-1.699	20.753	CENPE;CENPU;HELLS;CENPF;HJURP
positive regulation of cell cycle process (GO:0090068)	7/108	9.07E-06	7.42E-04	-1.248	14.487	INSR;KIF14;NUSAP1;HMGA2;KIF23;AURKB; CIT
regulation of mitotic nuclear division (GO:0007088)	6/85	2.49E-05	1.66E-03	-1.377	14.600	INSR;CHEK1;NUSAP1;KNTC1;KIF11;MKI67
complement activation, classical pathway (GO:0006958)	7/124	2.24E-05	1.66E-03	-1.068	11.436	IGLV3-25;IGHV3-21;IGHV4-34;IGHD;IGKV3-15; IGLV3-1;IGKV3-11

Table 2C (Continued)

Term	Overlap	P-value	Adjusted P-value	Z-score	Combined Score	Genes
humoral immune response mediated by circulating immunoglobulin (GO:0002455)	7/126	2.48E-05	1.66E-03	-1.036	10.988	IGLV3-25;IGHV3-21;IGHV4-34;IGHD;IGKV3-15; IGLV3-1;IGKV3-11
strand displacement (GO:0000732)	4/27	3.18E-05	1.99E-03	-2.577	26.685	BLM;BRIP1;RMI2;BRCA2
antigen processing and presentation of exogenous peptide antigen via MHC class II (GO:0019886)	6/98	5.58E-05	2.73E-03	-1.955	19.146	CENPE;HLA-DMA;KIF23;KIF11;HLA-DOA;KIF15
antigen processing and presentation of exogenous peptide antigen (GO:0002478)	6/98	5.58E-05	2.73E-03	-1.886	18.475	CENPE;HLA-DMA;KIF23;KIF11;HLA-DOA;KIF15
antigen processing and presentation of peptide antigen via MHC class II (GO:0002495)	6/99	5.91E-05	2.73E-03	-1.834	17.856	CENPE;HLA-DMA;KIF23;KIF11;HLA-DOA;KIF15
regulation of chromosome segregation (GO:0051983)	4/30	4.89E-05	2.73E-03	-1.708	16.954	RMI2;MKI67;BUB1;AURKB
mitotic cytokinesis (GO:0000281)	5/60	5.46E-05	2.73E-03	-1.393	13.672	ESPL1;NUSAP1;KIF23;BRCA2;CIT
regulation of immune response (GO:0050776)	9/252	5.78E-05	2.73E-03	-1.271	12.403	IGLV3-25;BTN2A2;BTN3A1;IGHV4-34;HLA-B; IGKV3-15;IGLV3-1;HLA-F;IGKV3-11
sister chromatid segregation (GO:0000819)	4/33	7.18E-05	3.06E-03	-2.019	19.264	TOP2A;ESPL1;KIFC1;NUSAP1
G2 DNA damage checkpoint (GO:0031572)	4/33	7.18E-05	3.06E-03	-1.660	15.843	BLM;CHEK1;HMGA2;DTL
DNA synthesis involved in DNA repair (GO:0000731)	5/66	8.65E-05	3.54E-03	-1.298	12.145	BLM;BRIP1;RMI2;BRCA2;DTL
positive regulation of cytokinesis (GO:0032467)	4/35	9.09E-05	3.58E-03	-1.596	14.853	KIF14;KIF23;AURKB;CIT
interferon-gamma-mediated signaling pathway (GO:0060333)	5/71	1.23E-04	4.66E-03	-1.295	11.659	HCK;STAT1;OAS3;HLA-B;HLA-F
regulation of mitotic sister chromatid separation (GO:0010965)	3/16	1.61E-04	5.37E-03	-2.425	21.173	CENPE;UBE2C;TACC3
regulation of mitotic metaphase/anaphase transition (GO:0030071)	3/16	1.61E-04	5.37E-03	-2.335	20.391	CENPE;ESPL1;UBE2C
cellular response to interferon-gamma (GO:0071346)	6/117	1.49E-04	5.37E-03	-1.955	17.224	HCK;STAT1;OAS3;HLA-B;LGALS9;HLA-F
DNA biosynthetic process (GO:0071897)	4/40	1.55E-04	5.37E-03	-1.947	17.082	BLM;BRIP1;RMI2;BRCA2

Table 2C (Continued)

Term	Overlap	P-value	Adjusted P-value	Z-score	Combined Score	Genes
positive regulation of cell division (GO:0051781)	4/42	1.87E-04	6.04E-03	-2.049	17.588	KIF14;KIF23;AURKB;CIT
mitotic spindle checkpoint (GO:0071174)	3/18	2.33E-04	6.70E-03	-2.006	16.783	CENPF;BUB1B;BUB1
mitotic spindle assembly checkpoint (GO:0007094)	3/18	2.33E-04	6.70E-03	-1.997	16.708	CENPF;BUB1B;BUB1
spindle assembly checkpoint (GO:0071173)	3/18	2.33E-04	6.70E-03	-1.967	16.453	CENPF;BUB1B;BUB1
anaphase-promoting complex-dependent catabolic process (GO:0031145)	5/80	2.16E-04	6.70E-03	-1.450	12.244	CDC20;UBE2C;PSME4;BUB1B;AURKB
metaphase plate congression (GO:0051310)	4/45	2.46E-04	6.87E-03	-1.392	11.568	CENPE;CENPF;KIFC1;KIF14
Fc-gamma receptor signaling pathway involved in phagocytosis (GO:0038096)	6/134	3.12E-04	8.50E-03	-1.183	9.550	HCK;IGLV3-25;IGHV4-34;IGKV3-15;IGLV3-1; IGKV3-11
Fc-gamma receptor signaling pathway (GO:0038094)	6/135	3.24E-04	8.63E-03	-2.012	16.163	HCK;IGLV3-25;IGHV4-34;IGKV3-15;IGLV3-1; IGKV3-11
Fc receptor mediated stimulatory signaling pathway (GO:0002431)	6/136	3.37E-04	8.76E-03	-1.160	9.272	HCK;IGLV3-25;IGHV4-34;IGKV3-15;IGLV3-1; IGKV3-11
regulation of signal transduction by p53 class mediator (GO:1901796)	6/139	3.79E-04	9.39E-03	-1.426	11.235	TPX2;BLM;BRIP1;RMI2;CHEK1;AURKB
response to cytokine (GO:0034097)	6/139	3.79E-04	9.39E-03	-1.246	9.818	STAT1;LAMP3;PTPN6;LGALS9;TNFRSF11A; JAK3
cytokine-mediated signaling pathway (GO:0019221)	13/634	3.94E-04	9.52E-03	-1.306	10.239	STAT1;HLA-B;TNFRSF11A;HLA-F;LMNB1;CXCL10; HCK;AIM2;OAS3;IL2RB;PSME4;PTPN6;JAK3
positive regulation of defense response to virus by host (GO:0002230)	3/22	4.31E-04	9.97E-03	-2.136	16.554	AIM2;DTX3L;STAT1
peptidyl-tyrosine modification (GO:0018212)	4/52	4.30E-04	9.97E-03	-1.799	13.946	HCK;INSR;PTPN6;JAK3
regulation of interferon-gamma-mediated signaling pathway (GO:0060334)	3/23	4.93E-04	1.12E-02	-2.154	16.405	STAT1;NLRC5;PARP14
chromosome condensation (GO:0030261)	3/24	5.61E-04	1.24E-02	-1.779	13.318	TOP2A;NUSAP1;NCAPH
DNA recombination (GO:0006310)	4/56	5.71E-04	1.24E-02	-1.569	11.716	BLM;BRIP1;RMI2;BRCA2
regulation of cell cycle G1/S phase transition (GO:1902806)	3/25	6.34E-04	1.35E-02	-2.125	15.646	E2F1;KIF14;PTPN6

Table 2C (Continued)

Term	Overlap	P-value	Adjusted P-value	Z-score	Combined Score	Genes
regulation of transcription involved in G1/S transition of mitotic cell cycle (GO:0000083)	3/27	7.98E-04	1.63E-02	-1.885	13.443	RRM2;ORC1;E2F1
G1/S transition of mitotic cell cycle (GO:0000082)	5/106	7.90E-04	1.63E-02	-1.165	8.319	RRM2;ORC1;E2F1;MCM10;MCM5
centromeric sister chromatid cohesion (GO:0070601)	2/7	9.43E-04	1.76E-02	-3.783	26.354	BUB1B;BUB1
histone-serine phosphorylation (GO:0035404)	2/7	9.43E-04	1.76E-02	-2.915	20.307	HMGA2;AURKB
regulation of protein activation cascade (GO:2000257)	5/109	8.96E-04	1.76E-02	-1.980	13.896	IGLV3-25;IGHV4-34;IGKV3-15;IGLV3-1;IGKV3-11
cellular macromolecule biosynthetic process (GO:0034645)	9/368	9.45E-04	1.76E-02	-1.686	11.743	BLM;BRIP1;RMI2;ORC1;CHEK1;E2F1;TIMELESS; MCM10;MCM5
regulation of complement activation (GO:0030449)	5/110	9.34E-04	1.76E-02	-1.316	9.183	IGLV3-25;IGHV4-34;IGKV3-15;IGLV3-1;IGKV3-11
antigen processing and presentation of peptide antigen via MHC class I (GO:0002474)	3/29	9.87E-04	1.81E-02	-1.708	11.823	HLA-B;TAP1;HLA-F
type I interferon signaling pathway (GO:0060337)	4/66	1.06E-03	1.85E-02	-2.220	15.200	STAT1;OAS3;HLA-B;HLA-F
regulation of humoral immune response (GO:0002920)	5/114	1.10E-03	1.85E-02	-2.174	14.821	IGLV3-25;IGHV4-34;IGKV3-15;IGLV3-1;IGKV3-11
apoptotic process (GO:0006915)	7/232	1.06E-03	1.85E-02	-1.946	13.328	ERBB3;ESPL1;CHEK1;GZMB;NOX5;PTPN6;CIT
positive regulation of interleukin-1 secretion (GO:0050716)	3/30	1.09E-03	1.85E-02	-1.721	11.737	AIM2;IGHD;LGALS9
cellular response to type I interferon (GO:0071357)	4/66	1.06E-03	1.85E-02	-1.347	9.223	STAT1;OAS3;HLA-B;HLA-F
regulation of immune effector process (GO:0002697)	5/115	1.14E-03	1.89E-02	-2.762	18.719	IGLV3-25;IGHV4-34;IGKV3-15;IGLV3-1;IGKV3-11
replication fork processing (GO:0031297)	3/31	1.20E-03	1.94E-02	-2.085	14.018	BLM;RFWD3;BRCA2
regulation of defense response to virus by host (GO:0050691)	3/31	1.20E-03	1.94E-02	-1.775	11.937	AIM2;DTX3L;STAT1

Table 2C (Continued)

Term	Overlap	P-value	Adjusted P-value	Z-score	Combined Score	Genes
cellular response to hydroxyurea (GO:0072711)	2/8	1.25E-03	1.96E-02	-3.156	21.095	BLM;TIMELESS
replication fork protection (GO:0048478)	2/8	1.25E-03	1.96E-02	-2.731	18.254	BLM;TIMELESS
response to hydroxyurea (GO:0072710)	2/9	1.60E-03	2.16E-02	-3.015	19.407	BLM;TIMELESS
regulation of metaphase/anaphase transition of cell cycle (GO:1902099)	2/9	1.60E-03	2.16E-02	-3.000	19.312	CENPE;UBE2C
heterochromatin assembly (GO:0031507)	2/9	1.60E-03	2.16E-02	-2.757	17.744	HELLS;HMGA2
kinetochore assembly (GO:0051382)	2/9	1.60E-03	2.16E-02	-2.750	17.700	CENPE;CENPF
interleukin-21-mediated signaling pathway (GO:0038114)	2/9	1.60E-03	2.16E-02	-2.684	17.275	STAT1;JAK3
chromatin remodeling at centromere (GO:0031055)	3/33	1.45E-03	2.16E-02	-2.212	14.465	CENPU;HELLS;HJURP
cellular response to interleukin-21 (GO:0098757)	2/9	1.60E-03	2.16E-02	-2.241	14.426	STAT1;JAK3
negative regulation of activated T cell proliferation (GO:0046007)	2/9	1.60E-03	2.16E-02	-2.199	14.157	BTN2A2;LGALS9
enzyme linked receptor protein signaling pathway (GO:0007167)	5/121	1.43E-03	2.16E-02	-1.681	11.013	HCK;ERBB3;CD7;BLNK;JAK3
regulation of acute inflammatory response (GO:0002673)	5/122	1.48E-03	2.16E-02	-1.509	9.832	IGLV3-25;IGHV4-34;IGKV3-15;IGLV3-1;IGKV3-11
Fc-epsilon receptor signaling pathway (GO:0038095)	6/183	1.59E-03	2.16E-02	-1.158	7.459	IGLV3-25;IGHV4-34;PSME4;IGKV3-15;IGLV3-1; IGKV3-11
Fc receptor signaling pathway (GO:0038093)	6/184	1.64E-03	2.18E-02	-1.271	8.156	IGLV3-25;IGHV4-34;PSME4;IGKV3-15;IGLV3-1; IGKV3-11
antigen processing and presentation of exogenous peptide antigen via MHC class I, TAP-dependent (GO:0002479)	4/76	1.80E-03	2.36E-02	-1.207	7.629	PSME4;HLA-B;TAP1;HLA-F
DNA-dependent DNA replication maintenance of fidelity (GO:0045005)	3/36	1.86E-03	2.40E-02	-1.883	11.835	BLM;RFWD3;TIMELESS
regulation of protein processing (GO:0070613)	5/129	1.89E-03	2.40E-02	-1.727	10.829	IGLV3-25;IGHV4-34;IGKV3-15;IGLV3-1;IGKV3-11

Table 2C (Continued)

Term	Overlap	P-value	Adjusted P-value	Z-score	Combined Score	Genes
positive regulation of ubiquitin-protein ligase activity involved in regulation of mitotic cell cycle transition (GO:0051437)	4/77	1.89E-03	2.40E-02	-1.403	8.800	CDC20;UBE2C;PSME4;BUB1B
antigen processing and presentation of exogenous peptide antigen via MHC class I, TAP-independent (GO:0002480)	2/10	1.99E-03	2.47E-02	-2.224	13.830	HLA-B;HLA-F
regulation of cytokinesis (GO:0032465)	4/78	1.98E-03	2.47E-02	-2.098	13.062	KIF14;KIF23;AURKB;CIT
antigen processing and presentation of exogenous peptide antigen via MHC class I (GO:0042590)	4/79	2.07E-03	2.53E-02	-1.214	7.502	PSME4;HLA-B;TAP1;HLA-F
regulation of single stranded viral RNA replication via double stranded DNA intermediate (GO:0045091)	2/11	2.43E-03	2.55E-02	-2.608	15.707	TOP2A;HMGA2
positive regulation of T cell apoptotic process (GO:0070234)	2/11	2.43E-03	2.55E-02	-2.510	15.114	LGALS9;IDO1
establishment of spindle localization (GO:0051293)	2/11	2.43E-03	2.55E-02	-2.278	13.720	ESPL1;NUSAP1
regulation of T cell apoptotic process (GO:0070232)	2/11	2.43E-03	2.55E-02	-2.142	12.897	JAK3;IDO1
mitotic DNA damage checkpoint (GO:0044773)	3/40	2.53E-03	2.55E-02	-2.114	12.643	BLM;RFWD3;HMGA2
regulation of type I interferon-mediated signaling pathway (GO:0060338)	3/40	2.53E-03	2.55E-02	-2.047	12.239	STAT1;NLRC5;PTPN6
mitotic spindle midzone assembly (GO:0051256)	2/11	2.43E-03	2.55E-02	-1.917	11.545	KIF23;AURKB
positive regulation of ubiquitin protein ligase activity (GO:1904668)	4/83	2.48E-03	2.55E-02	-1.903	11.415	CDC20;UBE2C;PSME4;BUB1B
spindle assembly (GO:0051225)	4/81	2.27E-03	2.55E-02	-1.864	11.348	TPX2;KIFC1;MYBL2;KIF11
retrograde vesicle-mediated transport, Golgi to ER (GO:0006890)	4/81	2.27E-03	2.55E-02	-1.693	10.305	CENPE;KIF23;KIF11;KIF15
endocytosis (GO:0006897)	7/264	2.22E-03	2.55E-02	-1.591	9.720	DENND1C;IGLV3-25;IGHV4-34;IGKV3-15; IGLV3-1;IGKV3-11;MERTK

Table 2C (Continued)

Term	Overlap	P-value	Adjusted P-value	Z-score	Combined Score	Genes
DNA damage checkpoint (GO:0000077)	3/39	2.35E-03	2.55E-02	-1.528	9.247	BRIP1;CHEK1;E2F1
negative regulation of G0 to G1 transition (GO:0070317)	3/38	2.18E-03	2.55E-02	-1.474	9.035	RRM2;CHEK1;E2F1
positive regulation of cell proliferation (GO:0008284)	9/425	2.53E-03	2.55E-02	-1.430	8.548	HCK;STAT1;INSR;SIRPG;E2F1;KIF14;HMGA2; PTPN6;TNFRSF11A
regulation of G0 to G1 transition (GO:0070316)	3/40	2.53E-03	2.55E-02	-1.411	8.437	RRM2;CHEK1;E2F1
peptidyl-tyrosine phosphorylation (GO:0018108)	4/80	2.17E-03	2.55E-02	-1.361	8.349	HCK;INSR;PTPN6;JAK3
DNA replication-independent nucleosome assembly (GO:0006336)	3/40	2.53E-03	2.55E-02	-1.391	8.317	CENPU;HJURP;ASF1B
negative regulation of cell cycle process (GO:0010948)	4/82	2.37E-03	2.55E-02	-1.331	8.043	RRM2;CHEK1;E2F1;AURKB
positive regulation of transferase activity (GO:0051347)	5/138	2.54E-03	2.55E-02	-1.145	6.839	DTX3L;ERBB3;INSR;MERTK;AURKB
positive regulation of mitotic cell cycle phase transition (GO:1901992)	3/41	2.71E-03	2.67E-02	-1.737	10.266	ESPL1;UBE2C;DTL
positive regulation of mitotic nuclear division (GO:0045840)	3/41	2.71E-03	2.67E-02	-1.603	9.475	UBE2C;INSR;NUSAP1
cellular response to interleukin-2 (GO:0071352)	2/12	2.90E-03	2.69E-02	-2.496	14.588	IL2RB;JAK3
negative regulation of cell division (GO:0051782)	2/12	2.90E-03	2.69E-02	-2.340	13.678	BLM;AURKB
mitotic spindle elongation (GO:0000022)	2/12	2.90E-03	2.69E-02	-2.326	13.596	KIF23;AURKB
regulation of mitotic centrosome separation (GO:0046602)	2/12	2.90E-03	2.69E-02	-2.301	13.448	CHEK1;KIF11
interleukin-2-mediated signaling pathway (GO:0038110)	2/12	2.90E-03	2.69E-02	-1.860	10.869	IL2RB;JAK3
double-strand break repair (GO:0006302)	5/142	2.88E-03	2.69E-02	-1.811	10.598	BLM;BRIP1;DTX3L;RFWD3;BRCA2
regulation of phagocytosis (GO:0050764)	3/42	2.91E-03	2.69E-02	-1.587	9.267	HCK;SIRPG;MERTK

Table 2C (Continued)

Term	Overlap	P-value	Adjusted P-value	Z-score	Combined Score	Genes
positive regulation of protein ubiquitination involved in ubiquitin-dependent protein catabolic process (GO:2000060)	4/88	3.07E-03	2.81E-02	-1.759	10.178	CDC20;UBE2C;PSME4;BUB1B
positive regulation of interleukin-1 production (GO:0032732)	2/13	3.41E-03	2.95E-02	-2.576	14.637	AIM2;IGHD
protein localization to kinetochore (GO:0034501)	2/13	3.41E-03	2.95E-02	-2.556	14.522	BUB1B;AURKB
kinetochore organization (GO:0051383)	2/13	3.41E-03	2.95E-02	-2.490	14.147	CENPE;CENPF
regulation of sister chromatid cohesion (GO:0007063)	2/13	3.41E-03	2.95E-02	-2.438	13.849	ESPL1;BUB1
signal transduction involved in G2 DNA damage checkpoint (GO:0072425)	2/13	3.41E-03	2.95E-02	-2.302	13.079	CHEK1;DTL
regulation of double-strand break repair via nonhomologous end joining (GO:2001032)	2/13	3.41E-03	2.95E-02	-2.055	11.675	DTX3L;HMGA2
mitotic metaphase plate congression (GO:0007080)	3/44	3.32E-03	2.95E-02	-1.723	9.832	CENPE;KIFC1;KIF14
regulation of cell cycle process (GO:0010564)	4/91	3.46E-03	2.97E-02	-1.449	8.209	RMI2;HMGA2;MKI67;AURKB
regulation of G2/M transition of mitotic cell cycle (GO:0010389)	5/150	3.64E-03	3.10E-02	-1.605	9.014	TPX2;CENPF;PSME4;KIF14;DTL
cellular response to interleukin-15 (GO:0071350)	2/14	3.96E-03	3.27E-02	-2.332	12.901	IL2RB;JAK3
establishment of chromosome localization (GO:0051303)	2/14	3.96E-03	3.27E-02	-2.237	12.374	CENPE;CENPF
signal transduction involved in DNA damage checkpoint (GO:0072422)	2/14	3.96E-03	3.27E-02	-2.193	12.129	CHEK1;DTL
interleukin-15-mediated signaling pathway (GO:0035723)	2/14	3.96E-03	3.27E-02	-1.575	8.714	IL2RB;JAK3
regulation of G1/S transition of mitotic cell cycle (GO:2000045)	3/47	4.01E-03	3.28E-02	-1.871	10.328	E2F1;KIF14;PTPN6
interstrand cross-link repair (GO:0036297)	3/49	4.51E-03	3.66E-02	-1.596	8.623	FANCD2;RFWD3;FANCA

Table 2C (Continued)

Term	Overlap	P-value	Adjusted P-value	Z-score	Combined Score	Genes
cellular response to nitrogen compound (GO:1901699)	2/15	4.55E-03	3.67E-02	-2.262	12.198	BLM;TIMELESS
protein localization to chromosome, centromeric region (GO:0071459)	2/16	5.17E-03	4.01E-02	-2.474	13.023	BUB1B;AURKB
negative regulation of DNA-dependent DNA replication (GO:2000104)	2/16	5.17E-03	4.01E-02	-2.081	10.957	BLM;TIMELESS
regulation of exit from mitosis (GO:0007096)	2/16	5.17E-03	4.01E-02	-2.003	10.544	UBE2C;KNTC1
mitotic chromosome condensation (GO:0007076)	2/16	5.17E-03	4.01E-02	-1.807	9.513	NUSAP1;NCAPH
positive regulation of double-strand break repair (GO:2000781)	2/16	5.17E-03	4.01E-02	-1.468	7.727	DTX3L;TIMELESS
regulation of kinase activity (GO:0043549)	4/102	5.20E-03	4.01E-02	-1.226	6.447	ERBB3;INSR;NLRC5;MERTK
B cell differentiation (GO:0030183)	3/53	5.62E-03	4.27E-02	-1.352	7.004	CD79A;BLNK;JAK3
regulation of DNA binding (GO:0051101)	3/53	5.62E-03	4.27E-02	-1.233	6.386	E2F1;HJURP;HMGA2
neuron apoptotic process (GO:0051402)	2/17	5.84E-03	4.41E-02	-2.167	11.148	ERBB3;CIT
positive regulation of phosphatidylinositol 3-kinase signaling (GO:0014068)	3/54	5.92E-03	4.44E-02	-1.451	7.442	ERBB3;PTPN6;PIK3AP1
cellular response to inorganic substance (GO:0071241)	2/18	6.54E-03	4.83E-02	-2.196	11.044	BLM;TIMELESS
mitotic G2 DNA damage checkpoint (GO:0007095)	2/18	6.54E-03	4.83E-02	-1.880	9.457	BLM;HMGA2
positive regulation of protein kinase activity (GO:0045860)	5/173	6.62E-03	4.86E-02	-1.231	6.177	CENPE;TPX2;ERBB3;INSR;KIF14
chromosome organization (GO:0051276)	3/57	6.88E-03	4.98E-02	-1.487	7.405	TOP2A;HMGA2;BRCA2
DNA-dependent DNA replication (GO:0006261)	3/57	6.88E-03	4.98E-02	-1.437	7.154	GINS1;ORC1;MCM10

Table 2D. Reactome pathways of the genes lower expressed with age

Term	Overlap	P-value	Adjusted P-value	Z-score	Combined Score	Genes
Cell Cycle_Homo sapiens_R-HSA-1640170	34/566	7.60E-23	3.40E-20	-2.461	125.347	TOP2A;BLM;HIST1H2BL;HJURP;CASC5;BUB1B; MCM10;BRCA2;NCAPH;AURKB;LMNB1;CDC20; BRIP1;ORC1;CHEK1;E2F1;MYBL2;KNTC1;WHSC1; BUB1;GINS1;CENPU;RMI2;RRM2;NUP210;UBE2C; HIST1H2AJ;KIF23;CENPE;TPX2;CENPF;ESPL1; PSME4;MCM5
Cell Cycle, Mitotic_Homo sapiens_R-HSA-69278	27/462	7.08E-18	1.58E-15	-2.468	97.462	TOP2A;HIST1H2BL;CASC5;BUB1B;MCM10; NCAPH;AURKB;LMNB1;CDC20;ORC1;E2F1; MYBL2;KNTC1;BUB1;GINS1;CENPU;RRM2; NUP210;UBE2C;HIST1H2AJ;KIF23;CENPE;TPX2; CENPF;ESPL1;PSME4;MCM5
M Phase_Homo sapiens_R-HSA-68886	18/268	3.26E-13	4.86E-11	-2.432	69.927	CENPU;NUP210;UBE2C;HIST1H2BL;HIST1H2AJ; BUB1B;CASC5;KIF23;NCAPH;AURKB;LMNB1; CDC20;CENPE;CENPF;ESPL1;PSME4;KNTC1; BUB1
Mitotic Anaphase_Homo sapiens_R-HSA-68882	13/173	1.85E-10	1.66E-08	-2.323	52.054	CENPU;UBE2C;BUB1B;CASC5;AURKB;LMNB1; CDC20;CENPE;CENPF;ESPL1;PSME4;KNTC1; BUB1
Mitotic Metaphase and Anaphase_Homo sapiens_R-HSA-2555396	13/174	1.99E-10	1.66E-08	-2.317	51.748	CENPU;UBE2C;BUB1B;CASC5;AURKB;LMNB1; CDC20;CENPE;CENPF;ESPL1;PSME4;KNTC1; BUB1
RHO GTPase Effectors_Homo sapiens_R-HSA-195258	15/255	2.22E-10	1.66E-08	-2.225	49.463	CENPU;HIST1H2BL;HIST1H2AJ;KIF14;BUB1B; CASC5;IQGAP3;AURKB;CIT;CDC20;CENPE; CENPF;DIAPH3;KNTC1;BUB1
Cell Cycle Checkpoints_Homo sapiens_R-HSA-69620	13/182	3.48E-10	2.23E-08	-2.316	50.445	BLM;RMI2;UBE2C;HIST1H2BL;BUB1B;MCM10; CDC20;BRIP1;ORC1;CHEK1;PSME4;MCM5; WHSC1
Signaling by Rho GTPases_Homo sapiens_R-HSA-194315	17/367	5.32E-10	2.98E-08	-2.149	45.885	ARHGAP11A;CENPU;HIST1H2BL;HIST1H2AJ; KIF14;BUB1B;CASC5;ARHGAP26;IQGAP3; AURKB;CIT;CDC20;CENPE;CENPF;DIAPH3; KNTC1;BUB1

Table 2D (Continued)

Term	Overlap	P-value	Adjusted P-value	Z-score	Combined Score	Genes
Separation of Sister Chromatids_Homo sapiens_R-HSA-2467813	12/162	1.13E-09	5.62E-08	-2.251	46.385	CDC20;CENPU;CENPE;CENPF;ESPL1;UBE2C; PSME4;BUB1B;CASC5;KNTC1;BUB1;AURKB
Mitotic Prometaphase_Homo sapiens_R-HSA-68877	10/107	3.04E-09	1.36E-07	-1.974	38.714	CDC20;CENPU;CENPE;CENPF;BUB1B;CASC5; KNTC1;BUB1;NCAPH;AURKB
RHO GTPases Activate Formins_Homo sapiens_R-HSA-5663220	10/114	5.65E-09	2.30E-07	-1.945	36.929	CDC20;CENPU;CENPE;CENPF;DIAPH3;BUB1B; CASC5;KNTC1;BUB1;AURKB
Immune System_Homo sapiens_R-HSA-168256	32/1547	9.71E-09	3.62E-07	-2.139	39.466	NLRC5;KIF11;TNFRSF11A;KIF15;CDC20;CD79A; IGLV3-25;HLA-DMA;ERBB3;BLNK;LGALS9; HLA-DOA;JAK3;NUP210;DTX3L;UBE2C;STAT1; INSR;HLA-B;TAP1;KIF23;HLA-F;CENPE;HCK; AIM2;OAS3;IL2RB;PSME4;IGHD;TLR10;PTPN6; PIK3AP1
Resolution of Sister Chromatid Cohesion_Homo sapiens_R-HSA-2500257	9/99	2.46E-08	8.46E-07	-1.987	34.808	CDC20;CENPU;CENPE;CENPF;BUB1B;CASC5; KNTC1;BUB1;AURKB
Adaptive Immune System_Homo sapiens_R-HSA-1280218	21/762	4.74E-08	1.52E-06	-2.173	36.643	DTX3L;UBE2C;INSR;HLA-B;TAP1;KIF23;KIF11; HLA-F;KIF15;CDC20;CENPE;IGLV3-25;CD79A; HLA-DMA;ERBB3;PSME4;IGHD;BLNK;PTPN6; HLA-DOA;PIK3AP1
G2/M Checkpoints_Homo sapiens_R-HSA-69481	10/150	7.90E-08	2.36E-06	-2.227	36.423	BLM;BRIP1;RMI2;ORC1;HIST1H2BL;CHEK1; PSME4;MCM5;MCM10;WHSC1
HDR through Homologous Recombination (HR) or Single Strand Annealing (SSA)_Homo sapiens_R-HSA-5693567	8/112	9.76E-07	2.73E-05	-1.914	26.496	BLM;BRIP1;RMI2;HIST1H2BL;CHEK1;TIMELESS; WHSC1;BRCA2
Processing of DNA double-strand break ends_Homo sapiens_R-HSA-5693607	7/81	1.32E-06	3.47E-05	-1.973	26.709	RMI2;BLM;BRIP1;HIST1H2BL;CHEK1;TIMELESS; WHSC1
Homology Directed Repair_Homo sapiens_R-HSA-5693538	8/118	1.45E-06	3.61E-05	-1.915	25.744	BLM;BRIP1;RMI2;HIST1H2BL;CHEK1;TIMELESS; WHSC1;BRCA2
DNA Repair_Homo sapiens_R-HSA-73894	11/285	4.09E-06	9.64E-05	-2.126	26.380	BRIP1;RMI2;BLM;FANCD2;HIST1H2BL;CHEK1; TIMELESS;FANCA;WHSC1;BRCA2;DTL
DNA Double-Strand Break Repair_Homo sapiens_R-HSA-5693532	8/145	6.77E-06	1.44E-04	-1.952	23.238	BLM;BRIP1;RMI2;HIST1H2BL;CHEK1;TIMELESS; WHSC1;BRCA2

Table 2D (Continued)

Term	Overlap	P-value	Adjusted P-value	Z-score	Combined Score	Genes
Presynaptic phase of homologous DNA pairing and strand exchange_Homo sapiens_R-HSA-5693616	5/39	6.46E-06	1.44E-04	-1.869	22.337	RMI2;BLM;BRIP1;CHEK1;BRCA2
Homologous DNA Pairing and Strand Exchange_Homo sapiens_R-HSA-5693579	5/42	9.39E-06	1.91E-04	-1.874	21.694	RMI2;BLM;BRIP1;CHEK1;BRCA2
Kinesins_Homo sapiens_R-HSA-983189	5/44	1.19E-05	2.31E-04	-1.683	19.093	CENPE;KIFC1;KIF23;KIF11;KIF15
G2/M DNA damage checkpoint_Homo sapiens_R-HSA-69473	6/78	1.52E-05	2.84E-04	-1.957	21.709	RMI2;BLM;BRIP1;HIST1H2BL;CHEK1;WHSC1
Antigen activates B Cell Receptor (BCR) leading to generation of second messengers_Homo sapiens_R-HSA-983695	5/47	1.65E-05	2.95E-04	-1.652	18.200	CD79A;IGHD;BLNK;PTPN6;PIK3AP1
Deposition of new CENPA-containing nucleosomes at the centromere_Homo sapiens_R-HSA-606279	5/52	2.72E-05	4.36E-04	-1.872	19.683	CENPU;HIST1H2BL;HIST1H2AJ;HJURP;CASC5
Nucleosome assembly_Homo sapiens_R-HSA-774815	5/52	2.72E-05	4.36E-04	-1.865	19.613	CENPU;HIST1H2BL;HIST1H2AJ;HJURP;CASC5
Resolution of D-loop Structures through Synthesis-Dependent Strand Annealing (SDSA)_Homo sapiens_R-HSA-5693554	4/26	2.72E-05	4.36E-04	-1.596	16.772	RMI2;BLM;BRIP1;BRCA2
Regulation of TP53 Activity through Phosphorylation_Homo sapiens_R-HSA-6804756	6/89	3.24E-05	5.00E-04	-1.684	17.404	TPX2;RMI2;BLM;BRIP1;CHEK1;AURKB
Mitotic G1-G1/S phases_Homo sapiens_R-HSA-453279	7/136	4.05E-05	6.05E-04	-1.892	19.135	TOP2A;RRM2;ORC1;E2F1;MYBL2;MCM5;MCM10
DNA Damage/Telomere Stress Induced Senescence_Homo sapiens_R-HSA-2559586	5/59	5.04E-05	7.28E-04	-1.708	16.906	HIST1H2BL;HIST1H2AJ;HMGA2;HIST1H1A; LMNB1
Resolution of D-loop Structures through Holliday Junction Intermediates_Homo sapiens_R-HSA-5693568	4/32	6.34E-05	8.88E-04	-1.616	15.620	RMI2;BLM;BRIP1;BRCA2
MHC class II antigen presentation_Homo sapiens_R-HSA-2132295	6/103	7.37E-05	9.55E-04	-1.669	15.882	CENPE;HLA-DMA;KIF23;KIF11;HLA-DOA;KIF15

Table 2D (Continued)

Term	Overlap	P-value	Adjusted P-value	Z-score	Combined Score	Genes
Resolution of D-Loop Structures_Homo sapiens_R-HSA-5693537	4/33	7.18E-05	9.55E-04	-1.653	15.772	RMI2;BLM;BRIP1;BRCA2
HDR through Homologous Recombination (HRR)_Homo sapiens_R-HSA-5685942	5/64	7.46E-05	9.55E-04	-1.638	15.567	RMI2;BLM;BRIP1;CHEK1;BRCA2
DNA Replication_Homo sapiens_R-HSA-69306	6/105	8.21E-05	1.02E-03	-1.989	18.709	GINS1;ORC1;PSME4;E2F1;MCM5;MCM10
Cytokine Signaling in Immune system_Homo sapiens_R-HSA-1280215	14/620	8.59E-05	1.04E-03	-2.084	19.516	NUP210;STAT1;HLA-B;TNFRSF11A;HLA-F;HCK; ERBB3;OAS3;PSME4;IL2RB;BLNK;PTPN6; LGALS9;JAK3
Interferon alpha/beta signaling_Homo sapiens_R-HSA-909733	5/68	9.98E-05	1.18E-03	-1.506	13.871	STAT1;OAS3;HLA-B;PTPN6;HLA-F
Activation of ATR in response to replication stress_Homo sapiens_R-HSA-176187	4/37	1.14E-04	1.30E-03	-1.525	13.849	ORC1;CHEK1;MCM5;MCM10
Cellular Senescence_Homo sapiens_R-HSA-2559583	7/161	1.18E-04	1.32E-03	-1.918	17.356	UBE2C;HIST1H2BL;HIST1H2AJ;E2F1;HMGA2; HIST1H1A;LMNB1
Regulation of DNA replication_Homo sapiens_R-HSA-69304	5/75	1.59E-04	1.74E-03	-1.930	16.883	ORC1;PSME4;E2F1;MCM5;MCM10
Signaling by the B Cell Receptor (BCR)_Homo sapiens_R-HSA-983705	8/233	1.97E-04	2.01E-03	-2.091	17.842	CD79A;ERBB3;INSR;PSME4;IGHD;BLNK;PTPN6; PIK3AP1
G1/S-Specific Transcription_Homo sapiens_R-HSA-69205	3/17	1.95E-04	2.01E-03	-1.016	8.679	RRM2;ORC1;E2F1
Formation of Senescence-Associated Heterochromatin Foci (SAHF)_Homo sapiens_R-HSA-2559584	3/17	1.95E-04	2.01E-03	-0.994	8.487	HMGA2;HIST1H1A;LMNB1
DNA Replication Pre-Initiation_Homo sapiens_R-HSA-69002	5/82	2.42E-04	2.31E-03	-1.867	15.543	ORC1;PSME4;E2F1;MCM5;MCM10
M/G1 Transition_Homo sapiens_R-HSA-68874	5/82	2.42E-04	2.31E-03	-1.858	15.467	ORC1;PSME4;E2F1;MCM5;MCM10
CD22 mediated BCR regulation_Homo sapiens_R-HSA-5690714	3/18	2.33E-04	2.31E-03	-0.850	7.107	CD79A;IGHD;PTPN6
APC/C-mediated degradation of cell cycle proteins_Homo sapiens_R-HSA-174143	5/85	2.86E-04	2.57E-03	-1.856	15.139	CDC20;UBE2C;PSME4;BUB1B;AURKB

Table 2D (Continued)

Term	Overlap	P-value	Adjusted P-value	Z-score	Combined Score	Genes
Regulation of mitotic cell cycle_Homo sapiens_R-HSA-453276	5/85	2.86E-04	2.57E-03	-1.836	14.981	CDC20;UBE2C;PSME4;BUB1B;AURKB
Meiosis_Homo sapiens_R-HSA-1500620	5/85	2.86E-04	2.57E-03	-1.583	12.916	BLM;HIST1H2BL;HIST1H2AJ;BRCA2;LMNB1
Chromosome Maintenance_Homo sapiens_R-HSA-73886	5/86	3.02E-04	2.66E-03	-1.625	13.166	CENPU;HIST1H2BL;HIST1H2AJ;HJURP;CASC5
Inactivation of APC/C via direct inhibition of the APC/C complex_Homo sapiens_R-HSA-141430	3/20	3.22E-04	2.72E-03	-0.879	7.067	CDC20;UBE2C;BUB1B
Inhibition of the proteolytic activity of APC/C required for the onset of anaphase by mitotic spindle checkpoint components_Homo sapiens_R-HSA-141405	3/20	3.22E-04	2.72E-03	-0.860	6.913	CDC20;UBE2C;BUB1B
Mitotic Spindle Checkpoint_Homo sapiens_R-HSA-69618	3/21	3.74E-04	3.10E-03	-0.918	7.247	CDC20;UBE2C;BUB1B
Interferon gamma signaling_Homo sapiens_R-HSA-877300	5/93	4.35E-04	3.54E-03	-1.333	10.319	STAT1;OAS3;HLA-B;PTPN6;HLA-F
Signal Transduction_Homo sapiens_R-HSA-162582	31/2465	4.66E-04	3.73E-03	-1.742	13.361	ARHGAP11A;HIST1H2BL;KIF14;CASC5;BUB1B; IQGAP3;AURKB;CDC20;ERBB3;CHEK1;E2F1; KNTC1;JAK3;BUB1;CD97;CENPU;STAT1;INSR; HIST1H2AJ;GZMB;MYO7A;ARHGAP26;CIT; CENPE;CXCL10;CENPF;KEL;DIAPH3;IL2RB; PSME4;PTPN6
Meiotic recombination_Homo sapiens_R-HSA-912446	4/54	4.97E-04	3.91E-03	-1.541	11.723	BLM;HIST1H2BL;HIST1H2AJ;BRCA2
Regulation of TP53 Activity_Homo sapiens_R-HSA-5633007	6/151	5.89E-04	4.55E-03	-1.564	11.630	TPX2;BLM;BRIP1;RMI2;CHEK1;AURKB
APC-Cdc20 mediated degradation of Nek2A_Homo sapiens_R-HSA-179409	3/25	6.34E-04	4.66E-03	-1.042	7.674	CDC20;UBE2C;BUB1B
G0 and Early G1_Homo sapiens_R-HSA-1538133	3/25	6.34E-04	4.66E-03	-0.903	6.648	TOP2A;E2F1;MYBL2

Table 2D (Continued)

Term	Overlap	P-value	Adjusted P-value	Z-score	Combined Score	Genes
Antigen Presentation: Folding, assembly and peptide loading of class I MHC_Homo sapiens_R-HSA-983170	3/25	6.34E-04	4.66E-03	-0.861	6.341	HLA-B;TAP1;HLA-F
Cellular responses to stress_Homo sapiens_R-HSA-2262752	9/367	9.27E-04	6.70E-03	-1.847	12.902	NUP210;UBE2C;HIST1H2BL;HIST1H2AJ;E2F1; HMGA2;NOX5;HIST1H1A;LMNB1
G1/S Transition_Homo sapiens_R-HSA-69206	5/112	1.01E-03	7.09E-03	-1.604	11.060	RRM2;ORC1;E2F1;MCM5;MCM10
ER-Phagosome pathway_Homo sapiens_R-HSA-1236974	4/65	1.00E-03	7.09E-03	-1.520	10.495	PSME4;HLA-B;TAP1;HLA-F
Activation of the pre-replicative complex_Homo sapiens_R-HSA-68962	3/30	1.09E-03	7.52E-03	-0.954	6.505	ORC1;MCM5;MCM10
Assembly of the pre-replicative complex_Homo sapiens_R-HSA-68867	4/67	1.13E-03	7.64E-03	-1.630	11.065	ORC1;PSME4;E2F1;MCM5
Signaling by Interleukins_Homo sapiens_R-HSA-449147	9/392	1.46E-03	9.03E-03	-1.864	12.168	HCK;ERBB3;STAT1;PSME4;IL2RB;BLNK;PTPN6; LGALS9;JAK3
Hemostasis_Homo sapiens_R-HSA-109582	11/552	1.40E-03	9.03E-03	-1.619	10.641	CENPE;ABCC4;KIFC1;IL2RB;SIRPG;PTPN6;KIF23; KIF11;MERTK;JAK3;KIF15
APC/C:Cdh1 mediated degradation of Cdc20 and other APC/C:Cdh1 targeted proteins in late mitosis/early G1_Homo sapiens_R-HSA-174178	4/71	1.40E-03	9.03E-03	-1.613	10.604	CDC20;UBE2C;PSME4;AURKB
Cdc20:Phospho-APC/C mediated degradation of Cyclin A_Homo sapiens_R-HSA-174184	4/71	1.40E-03	9.03E-03	-1.565	10.285	CDC20;UBE2C;PSME4;BUB1B
APC:Cdc20 mediated degradation of cell cycle proteins prior to satisfation of the cell cycle checkpoint_Homo sapiens_R-HSA-179419	4/72	1.47E-03	9.03E-03	-1.558	10.164	CDC20;UBE2C;PSME4;BUB1B
Removal of licensing factors from origins_Homo sapiens_R-HSA-69300	4/72	1.47E-03	9.03E-03	-1.553	10.129	ORC1;PSME4;MCM5;MCM10
E2F mediated regulation of DNA replication_Homo sapiens_R-HSA-113510	3/33	1.45E-03	9.03E-03	-1.029	6.730	RRM2;ORC1;E2F1
APC/C:Cdc20 mediated degradation of mitotic proteins_Homo sapiens_R-HSA-176409	4/74	1.63E-03	9.86E-03	-1.552	9.965	CDC20;UBE2C;PSME4;BUB1B

Table 2D (Continued)

Term	Overlap	P-value	Adjusted P-value	Z-score	Combined Score	Genes
Activation of APC/C and APC/C:Cdc20 mediated degradation of mitotic proteins_Homo sapiens_R-HSA-176814	4/75	1.71E-03	1.02E-02	-1.509	9.615	CDC20;UBE2C;PSME4;BUB1B
Signaling by SCF-KIT_Homo sapiens_R-HSA-1433557	8/325	1.74E-03	1.02E-02	-1.877	11.925	ERBB3;STAT1;INSR;CHEK1;PSME4;IL2RB; PTPN6;JAK3
HDR through Single Strand Annealing (SSA)_Homo sapiens_R-HSA-5685938	3/37	2.02E-03	1.17E-02	-1.002	6.216	RMI2;BLM;BRIP1
Interleukin-3, 5 and GM-CSF signaling_Homo sapiens_R-HSA-512988	7/261	2.08E-03	1.18E-02	-1.827	11.283	HCK;ERBB3;PSME4;IL2RB;BLNK;PTPN6;JAK3
Regulation of APC/C activators between G1/S and early anaphase_Homo sapiens_R-HSA-176408	4/79	2.07E-03	1.18E-02	-1.503	9.290	CDC20;UBE2C;PSME4;BUB1B
Interferon Signaling_Homo sapiens_R-HSA-913531	6/196	2.25E-03	1.26E-02	-1.448	8.832	NUP210;STAT1;OAS3;HLA-B;PTPN6;HLA-F
COPI-dependent Golgi-to-ER retrograde traffic_Homo sapiens_R-HSA-6811434	4/81	2.27E-03	1.26E-02	-1.193	7.264	CENPE;KIF23;KIF11;KIF15
Antigen processing-Cross presentation_Homo sapiens_R-HSA-1236975	4/82	2.37E-03	1.30E-02	-1.358	8.206	PSME4;HLA-B;TAP1;HLA-F
CDC6 association with the ORC:origin complex_Homo sapiens_R-HSA-68689	2/11	2.43E-03	1.31E-02	1.671	-10.064	ORC1;E2F1
Transcriptional Regulation by TP53_Homo sapiens_R-HSA-3700989	8/348	2.65E-03	1.42E-02	-1.613	9.566	TPX2;BRIP1;RMI2;BLM;FANCD2;CHEK1; E2F1;AURKB
Factors involved in megakaryocyte development and platelet production_Homo sapiens_R-HSA-983231	5/141	2.79E-03	1.47E-02	-1.317	7.746	CENPE;KIFC1;KIF23;KIF11;KIF15
Endosomal/Vacuolar pathway_Homo sapiens_R-HSA-1236977	2/12	2.90E-03	1.49E-02	1.307	-7.638	HLA-B;HLA-F
Unwinding of DNA_Homo sapiens_R-HSA-176974	2/12	2.90E-03	1.49E-02	1.753	-10.246	GINS1;MCM5

Table 2D (Continued)

Term	Overlap	P-value	Adjusted P-value	Z-score	Combined Score	Genes
SUMOylation of DNA replication proteins_Homo sapiens_R-HSA-4615885	3/44	3.32E-03	1.69E-02	-1.145	6.535	TOP2A;NUP210;AURKB
Signal regulatory protein (SIRP) family interactions_Homo sapiens_R-HSA-391160	2/13	3.41E-03	1.72E-02	0.896	-5.090	SIRPG;PTPN6
Regulation of IFNG signaling_Homo sapiens_R-HSA-877312	2/14	3.96E-03	1.97E-02	0.715	-3.955	STAT1;PTPN6
SUMO E3 ligases SUMOylate target proteins_Homo sapiens_R-HSA-3108232	4/96	4.19E-03	2.06E-02	-1.227	6.719	TOP2A;BLM;NUP210;AURKB
Synthesis of DNA_Homo sapiens_R-HSA-69239	4/97	4.35E-03	2.12E-02	-1.454	7.905	GINS1;ORC1;PSME4;MCM5
Association of licensing factors with the pre-replicative complex_Homo sapiens_R-HSA-69298	2/15	4.55E-03	2.19E-02	0.352	-1.898	ORC1;E2F1
Class I MHC mediated antigen processing & presentation_Homo sapiens_R-HSA-983169	7/305	4.91E-03	2.34E-02	-1.446	7.687	CDC20;DTX3L;UBE2C;PSME4;HLA-B;TAP1; HLA-F
Apoptosis_Homo sapiens_R-HSA-109581	5/163	5.17E-03	2.37E-02	-1.357	7.142	PSME4;E2F1;GZMB;HIST1H1A;LMNB1
SUMOylation_Homo sapiens_R-HSA-2990846	4/101	5.02E-03	2.37E-02	-1.176	6.228	TOP2A;BLM;NUP210;AURKB
Polo-like kinase mediated events_Homo sapiens_R-HSA-156711	2/16	5.17E-03	2.37E-02	0.494	-2.600	CENPF;MYBL2
RHO GTPases activate CIT_Homo sapiens_R-HSA-5625900	2/16	5.17E-03	2.37E-02	0.502	-2.642	KIF14;CIT
Programmed Cell Death_Homo sapiens_R-HSA-5357801	5/166	5.58E-03	2.52E-02	-1.352	7.018	PSME4;E2F1;GZMB;HIST1H1A;LMNB1
GPVI-mediated activation cascade_Homo sapiens_R-HSA-114604	3/53	5.62E-03	2.52E-02	-0.818	4.239	IL2RB;PTPN6;JAK3
Mitotic Prophase_Homo sapiens_R-HSA-68875	4/107	6.15E-03	2.73E-02	-1.239	6.305	NUP210;HIST1H2BL;HIST1H2AJ;LMNB1
Regulation of signaling by CBL_Homo sapiens_R-HSA-912631	2/18	6.54E-03	2.87E-02	0.330	-1.659	HCK;BLNK
Golgi-to-ER retrograde transport_Homo sapiens_R-HSA-8856688	4/110	6.78E-03	2.95E-02	-1.114	5.563	CENPE;KIF23;KIF11;KIF15

Table 2D (Continued)

Term	Overlap	P-value	Adjusted P-value	Z-score	Combined Score	Genes
Mitotic G2-G2/M phases_Homo sapiens_R-HSA-453274	5/175	6.94E-03	2.96E-02	-1.341	6.665	TPX2;CENPF;PSME4;E2F1;MYBL2
Meiotic synapsis_Homo sapiens_R-HSA-1221632	3/57	6.88E-03	2.96E-02	-0.889	4.425	HIST1H2BL;HIST1H2AJ;LMNB1
Conversion from APC/C:Cdc20 to APC/C:Cdh1 in late anaphase_Homo sapiens_R-HSA-176407	2/19	7.28E-03	3.08E-02	0.382	-1.879	CDC20;UBE2C
Interleukin-2 signaling_Homo sapiens_R-HSA-451927	6/252	7.60E-03	3.18E-02	-1.555	7.589	ERBB3;PSME4;IL2RB;PTPN6;LGALS9;JAK3
S Phase_Homo sapiens_R-HSA-69242	4/124	1.02E-02	4.23E-02	-1.300	5.956	GINS1;ORC1;PSME4;MCM5
APC/C:Cdc20 mediated degradation of Securin_Homo sapiens_R-HSA-174154	3/66	1.03E-02	4.23E-02	-1.160	5.307	CDC20;UBE2C;PSME4
APC/C:Cdc20 mediated degradation of Cyclin B_Homo sapiens_R-HSA-174048	2/23	1.06E-02	4.31E-02	-0.092	0.421	CDC20;UBE2C
Growth hormone receptor signaling_Homo sapiens_R-HSA-982772	2/24	1.15E-02	4.64E-02	0.066	-0.297	STAT1;PTPN6
Orc1 removal from chromatin_Homo sapiens_R-HSA-68949	3/70	1.21E-02	4.79E-02	-1.090	4.812	ORC1;PSME4;MCM5
Switching of origins to a post-replicative state_Homo sapiens_R-HSA-69052	3/70	1.21E-02	4.79E-02	-1.078	4.760	ORC1;PSME4;MCM5
Regulation of IFNA signaling_Homo sapiens_R-HSA-912694	2/25	1.24E-02	4.89E-02	0.143	-0.627	STAT1;PTPN6

Supplementary Table 3. Gene ontology and pathway analyses using g:Profiler

Table 3A. Biological processes of the genes higher expressed with age

Term	Overlap	FDR	Genes
homophilic cell adhesion via plasma membrane adhesion molecules (GO:0007156)	16/159	1.52E-11	CDH19;PCDHB4;PCDH11X;PCDHB6;PCDHB10;PCDHB14;PCDHB12; AMI(GO2;FAT3;PCDHB9;PCDH9;ROBO2;PCDHB13;TENM3;PCDHGA5;PCDHGA4
trans-synaptic signaling (GO:0099537)	24/613	4.28E-10	PCDHB4;RASD2;TRIM9;SLC8A3;RIMS4;LIN7A;PCDHB6;KCNIP2; PCDHB10;PCDHB14;PCDHB12;TMOD2;KCNMB4;SYT8;DLG2; SLC24A2;KIT;GRIK3;CACNB2;HAP1;RIMS2;PCDHB9;NRXN1;PCDHB13
synapse assembly (GO:0007416)	12/150	7.83E-08	PCDHB4;PCDHB6;PCDHB10;PCDHB14;PCDHB12;SLITRK3; EPHA7;GPM6A;PCDHB9;NRXN1;ROBO2;PCDHB13
regulation of neurotransmitter levels (GO:0001505)	11/304	5.26E-04	CPS1;NAALAD2;TRIM9;RIMS4;LIN7A;KCNMB4;HDC;SYT8; PRIMA1;RIMS2;NRXN1
neurotransmitter secretion (GO:0007269)	7/135	2.72E-03	TRIM9;RIMS4;LIN7A;KCNMB4;SYT8;RIMS2;NRXN1
synaptic vesicle localization (GO:0097479)	6/121	9.32E-03	TRIM9;RIMS4;LIN7A;SYT8;RIMS2;NRXN1
muscle system process (GO:0003012)	11/465	1.25E-02	MYOC;SLC8A3;KCNIP2;TMOD2;TNNI2;TMOD1;PDE5A; PRKCA;FBXO32;KIT;CACNB2
creatinine metabolic process (GO:0046449)	2/4	1.53E-02	GHR;MME
axon guidance (GO:0007411)	7/227	3.20E-02	NRP2;EPHA7;NCAM1;PRKCA;NRXN1;ROBO2;L1CAM
positive regulation of protein kinase B signaling (GO:0051897)	6/168	3.37E-02	MYOC;RASD2;FGF9;ANGPT1;KIT;NRXN1
hypothalamus cell differentiation (GO:0021979)	2/7	3.51E-02	NRP2;HAP1
pointed-end actin filament capping (GO:0051694)	2/7	3.51E-02	TMOD2;TMOD1
calcium ion-regulated exocytosis of neurotransmitter (GO:0048791)	3/30	3.66E-02	RIMS4;SYT8;RIMS2
regulation of calcium ion-dependent exocytosis (GO:0017158)	4/70	4.04E-02	TRIM9;RIMS4;SYT8;RIMS2
negative regulation of supramolecular fiber organization (GO:1902904)	5/127	4.71E-02	MYOC;TMOD2;TMOD1;TMEFF2;PLEKHH2
regulation of membrane potential (GO:0042391)	9/414	4.71E-02	MYOC;SLC8A3;RIMS4;KCNIP2;KCNMB4;GRIK3;CACNB2; RIMS2;NRXN1
synaptic vesicle cycle (GO:0099504)	5/128	4.75E-02	TRIM9;RIMS4;SYT8;RIMS2;NRXN1
blood circulation (GO:0008015)	10/501	4.75E-02	CPS1;SLC8A3;KCNIP2;TNNI2;NTS;KCNMB4;PDE5A;ANGPT1; CACNB2;MME

Table 3B. Reactome pathways of the genes higher expressed with age

Term	Overlap	FDR	Genes
Muscle contraction (R-HSA-397014)	8/200	1.06E-02	TBX5;SLC8A3;KCNIP2;TMOD2;TNNI2;TMOD1;CACNA2D3;CACNB2
cGMP effects (R-HSA-418457)	3/18	2.77E-02	KCNMB4;PRKG2;PDE5A
Neuronal System (R-HSA-112316)	9/365	3.42E-02	LIN7A;SLITRK3;KCNMB4;DLG2;PRKCA;CACNA2D3;GRIK3;CACNB2;NRXN1

Table 3C. Biological processes of the genes lower expressed with age

Term	Overlap	FDR	Genes
mitotic cell cycle process (GO:1903047)	36/776	2.27E-17	TACC3;MCM10;ORC1;TPX2;MCM5;GINS1;MYBL2;E2F1;PTPN6;CDC20;CENPF; KIF14;NCAPH;CIT;TOP2A;ESPL1;NUSAP1;KIF23;KIF11;CENPE;BRCA2;DTL; MKI67;CHEK1;HMGA2;BUB1B;RFWD3;BUB1;INSR;RRM2;UBE2C;AURKB; IQ-GAP3;KNTC1;BLM;KIFC1
nuclear division (GO:0000280)	27/401	1.02E-16	TACC3;ASPM;TPX2;MYBL2;CDC20;CENPF;KIF14;NCAPH;TOP2A;ESPL1;BRIP1; NUSAP1;KIF23;KIF11;CENPE;BRCA2;FANCD2;MKI67;CHEK1;BUB1B;BUB1; INSR;UBE2C;AURKB; KNTC1;FANCA;KIFC1
regulation of cell cycle process (GO:0010564)	28/706	5.06E-12	TACC3;TPX2;E2F1;PTPN6;CDC20;CENPF;KIF14;CIT;ESPL1;NUSAP1;KIF23; KIF11;CENPE;BRCA2;DTL;MKI67;CHEK1;HMGA2;BUB1B;RFWD3;BUB1;INSR; RRM2;UBE2C;RMI2; AURKB;KNTC1;BLM
cell proliferation (GO:0008283)	38/2121	5.45E-07	TACC3;BTN3A1;MCM10;ERBB3;ASPM;TPX2;SIRPG;GINS1;HCK;E2F1;CD79A; JAK3;PTPN6;STAT1;CDC20;CENPF;KIF14;HELLS;BTN2A2;ABCC4;IDO1;BRIP1; BRCA2;TNFRSF11A;MKI67;CHEK1;HMGA2;BUB1B;KIF15;LGALS9;CXCL10; BUB1;INSR;MARCKSL1;AURKB;IQGAP3;FANCA;NOX5
immune system process (GO:0002376)	47/3044	5.45E-07	BTN3A1;LAMP3;SIRPG;BLNK;GZMB;HCK;CD79A;JAK3;OAS3;PTPN6;STAT1; HELLS;CD97;BTN2A2;IDO1;TOP2A;KIF23;KIF11;CENPE;BRCA2;NLRC5; TNFRSF11A;FANCD2;MERTK;PIK3AP1;AIM2;KIF15;DTX3L;TAP1;LGALS9; CXCL10;PARP14;CD7;TLR10;FANCA; HSH2D; HLA-DOA;HLA-DMA;HLA-F; IGLV3-25;IGLV3-1;IGHD;IGHV3-21;IGHV4-34; HLA-B;IGKV3-11;IGKV3-15
meiotic nuclear division (GO:0140013)	11/173	1.75E-06	ASPM;CDC20;NCAPH;TOP2A;ESPL1;BRIP1;BRCA2;FANCD2;BUB1B; BUB1;FANCA
regulation of defense response (GO:0031347)	20/738	5.05E-06	HCK;PTPN6;STAT1;IDO1;NLRC5;TNFRSF11A;FANCD2;PIK3AP1;AIM2;DTX3L; LGALS9;PARP14;TLR10;FANCA;IGLV3-25;IGLV3-1;IGHV4-34;HLA-B; IGKV3-11; IGKV3-15
multi-organism process (GO:0051704)	38/2424	1.04E-05	ASPM;PSME4;LAMP3;IL2RB;HCK;E2F1;ASF1B;OAS3;STAT1;NCAPH; HIST1H1A;IDO1;TOP2A;NUP210;BRIP1;BRCA2;DIAPH3;NLRC5;TNFRSF11A; FANCD2;HMGA2;CENPU;MERTK;AIM2;DTX3L;LPO;TAP1;LGALS9;CXCL10; BUB1;INSR;SPATA32;FANCA;IGHD; IGHV3-21;HLA-B;KIFC1;NOX5
DNA replication (GO:0006260)	12/267	1.07E-05	MCM10;ORC1;MCM5;GINS1;BRIP1;BRCA2;DTL;CHEK1;RFWD3;RRM2; RMI2;BLM
interferon-gamma-mediated signaling pathway (GO:0060333)	7/85	5.56E-05	HCK;OAS3;STAT1;NLRC5;PARP14;HLA-F;HLA-B

Table 3C (Continued)

Term	Overlap	FDR	Genes
centromere complex assembly (GO:0034508)	5/52	5.63E-04	CENPF;HELLS;HJURP;CENPE;CENPU
DNA damage checkpoint (GO:0000077)	7/148	1.16E-03	E2F1;BRIP1;DTL;CHEK1;HMGA2;RFWD3;BLM
inner cell mass cell proliferation (GO:0001833)	3/12	1.18E-03	GINS1;BRCA2;CHEK1
leukocyte migration (GO:0050900)	12/464	1.19E-03	SIRPG;HCK;PTPN6;TNFRSF11A;MERTK;LGALS9;CXCL10;IGLV3-25;IGLV3-1; IGHV4-34;IGKV3-11;IGKV3-15
phagocytosis (GO:0006909)	10/329	1.31E-03	HCK;MYO7A;MERTK;IGLV3-25;IGLV3-1;IGHD;IGHV3-21;IGHV4-34;IGKV3-11; IGKV3-15
anaphase-promoting complex-dependent catabolic process (GO:0031145)	4/35	1.51E-03	CDC20;BUB1B;UBE2C;AURKB
regulation of interleukin-12 production (GO:0032655)	4/51	5.09E-03	JAK3;IDO1;LGALS9;HLA-B
negative regulation of T cell proliferation (GO:0042130)	4/55	6.23E-03	PTPN6;BTN2A2;IDO1;LGALS9
natural killer cell mediated cytotoxicity (GO:0042267)	4/55	6.23E-03	GZMB;PTPN6;LGALS9;HLA-B
positive regulation of defense response to virus by host (GO:0002230)	3/24	6.53E-03	STAT1;AIM2;DTX3L
metaphase plate congression (GO:0051310)	4/57	6.90E-03	CENPF;KIF14;CENPE;KIFC1
regulation of complement activation (GO:0030449)	5/105	7.93E-03	IGLV3-25;IGLV3-1;IGHV4-34;IGKV3-11;IGKV3-15
cellular response to hydroxyurea (GO:0072711)	2/7	9.84E-03	TIMELESS;BLM
positive regulation of interleukin-1 secretion (GO:0050716)	3/29	9.96E-03	AIM2;LGALS9;IGHD
regulation of regulatory T cell differentiation (GO:0045589)	3/29	9.96E-03	FANCD2;LGALS9;FANCA
respiratory burst (GO:0045730)	3/32	1.21E-02	HCK;CD52;INSR
regulation of single stranded viral RNA replication via double stranded DNA intermediate (GO:0045091)	2/9	1.45E-02	TOP2A;HMGA2
regulation of transferase activity (GO:0051338)	15/961	1.62E-02	ERBB3;TPX2;PTPN6;CDC20;KIF14;CENPE;NLRC5;TNFRSF11A;DTX3L;CXCL10; INSR;UBE2C;AURKB;IQGAP3;BLM
histone phosphorylation (GO:0016572)	3/37	1.71E-02	CHEK1;HMGA2;AURKB

Table 3C (Continued)

Term	Overlap	FDR	Genes
retrograde vesicle-mediated transport, Golgi to ER (GO:0006890)	4/83	1.99E-02	KIF23;KIF11;CENPE;KIF15
positive regulation of ubiquitin protein ligase activity (GO:1904668)	2/13	2.56E-02	CDC20;UBE2C
prostaglandin secretion (GO:0032310)	2/13	2.56E-02	ABCC4;TNFRSF11A
B cell receptor signaling pathway (GO:0050853)	4/103	3.59E-02	CD79A;PTPN6;IGHD;IGHV3-21
peptidyl-tyrosine autophosphorylation (GO:0038083)	3/53	3.77E-02	HCK;JAK3;INSR
regulation of DNA-binding transcription factor activity (GO:0051090)	8/406	4.01E-02	HCK;NLRC5;TNFRSF11A;FANCD2;HMGA2;AIM2;LGALS9;FANCA
kynurenic acid metabolic process (GO:0034275)	1/1	4.25E-02	IDO1
protein localization to kinetochore (GO:0034501)	2/18	4.25E-02	BUB1B;AURKB
mesodermal-endodermal cell signaling (GO:0003131)	1/1	4.25E-02	HMGA2
regulation of mammary gland epithelial cell proliferation (GO:0033599)	2/18	4.25E-02	BRCA2;IQGAP3
negative regulation of FasL biosynthetic process (GO:0045221)	1/1	4.25E-02	JAK3
cerebellar granular layer structural organization (GO:0021685)	1/1	4.25E-02	KIF14
thiocyanate metabolic process (GO:0018969)	1/1	4.25E-02	LPO
negative regulation of metanephric nephron tubule epithelial cell differentiation (GO:0072308)	1/1	4.25E-02	STAT1
lateral attachment of mitotic spindle microtubules to kinetochore (GO:0099607)	1/1	4.25E-02	CENPE
negative regulation by virus of viral protein levels in host cell (GO:0046725)	1/1	4.25E-02	STAT1
female sex differentiation (GO:0046660)	4/112	4.25E-02	BRCA2;MERTK;INSR;FANCA
toll-like receptor 10 signaling pathway (GO:0034166)	1/1	4.25E-02	TLR10

Table 3D. Reactome pathways of the genes lower expressed with age

Term	Overlap	FDR	Genes
Cell Cycle (R-HSA-1640170)	32/628	5.52E-13	MCM10;PSME4;ORC1;TPX2;MCM5;GINS1;MYBL2;E2F1;LMNB1;CDC20; CENPF;NCAPH;HJURP;TOP2A;NUP210;ESPL1;BRIP1;KIF23;CENPE;BRCA2; CHEK1;CENPU;BUB1B;BUB1;RRM2;UBE2C;RMI2;AURKB;KNTC1; HIST1H2BL;BLM;HIST1H2AJ
Antigen activates B Cell Receptor (BCR) leading to generation of second messengers (R-HSA-983695)	10/89	1.32E-06	BLNK;CD79A;PTPN6;PIK3AP1;IGLV3-25;IGLV3-1;IGHD;IGHV4-34; IGKV3-11;IGKV3-15
RHO GTPase Effectors (R-HSA-195258)	14/296	3.09E-05	CDC20;CENPF;KIF14;CIT;CENPE;DIAPH3;CENPU;BUB1B;BUB1;AURKB; IQ-GAP3;KNTC1;HIST1H2BL;HIST1H2AJ
Presynaptic phase of homolo (GOus DNA pairing and strand exchange (R-HSA-5693616)	5/39	7.40E-04	BRIP1;BRCA2;CHEK1;RMI2;BLM
FCGR activation (R-HSA-2029481)	6/67	8.98E-04	HCK;IGLV3-25;IGLV3-1;IGHV4-34;IGKV3-11;IGKV3-15
Kinesins (R-HSA-983189)	5/42	9.35E-04	KIF23;KIF11;CENPE;KIF15;KIFC1
Interleukin-3,5 and GM-CSF signaling (R-HSA-512988)	5/44	1.09E-03	BLNK;IL2RB;HCK;JAK3;PTPN6
Interleukin-2 family signaling (R-HSA-451927)	5/44	1.09E-03	IL2RB;JAK3;PTPN6;STAT1;LGALS9
DNA Replication (R-HSA-69306)	7/127	3.45E-03	MCM10;PSME4;ORC1;MCM5;GINS1;E2F1;UBE2C
Regulation of TP53 Activity through Phosphorylation (R-HSA-6804756)	6/92	3.88E-03	TPX2;BRIP1;CHEK1;RMI2;AURKB;BLM
Classical antibody-mediated complement activation (R-HSA-173623)	5/61	4.12E-03	IGLV3-25;IGLV3-1;IGHV4-34;IGKV3-11;IGKV3-15
Formation of Senescence-Associated Heterochromatin Foci (SAHF) (R-HSA-2559584)	3/16	5.15E-03	LMNB1;HIST1H1A;HMGA2
Role of LAT2/NTAL/LAB on calcium mobilization (R-HSA-2730905)	5/66	5.15E-03	IGLV3-25;IGLV3-1;IGHV4-34;IGKV3-11;IGKV3-15
Scavenging of heme from plasma (R-HSA-2168880)	5/67	5.15E-03	IGLV3-25;IGLV3-1;IGHV4-34;IGKV3-11;IGKV3-15
Cell surface interactions at the vascular wall (R-HSA-202733)	8/190	5.26E-03	SIRPG;PTPN6;MERTK;IGLV3-25;IGLV3-1;IGHV4-34;IGKV3-11;IGKV3-15
COPI-dependent golgi-to-ER retrograde traffic (R-HSA-6811434)	5/81	9.16E-03	KIF23;KIF11;CENPE;KIF15;KIFC1

Table 3D (Continued)

Term	Overlap	FDR	Genes
Antigen Presentation: Folding; assembly and peptide loading of class I MHC (R-HSA-983170)	3/24	1.17E-02	TAP1;HLA-F;HLA-B
Transcriptional Regulation by E2F6 (R-HSA-8953750)	3/34	2.45E-02	E2F1;CHEK1;RRM2
Endosomal/Vacuolar pathway (R-HSA-1236977)	2/11	2.65E-02	HLA-F;HLA-B
Deposition of new CENPA-containing nucleosomes at the centromere (R-HSA-606279)	4/73	2.68E-02	HJURP;CENPU;HIST1H2BL;HIST1H2AJ
Unwinding of DNA (R-HSA-176974)	2/12	2.75E-02	MCM5;GINS1
Signal regulatory protein family interactions (R-HSA-391160)	2/13	3.13E-02	SIRPG;PTPN6
Signaling by SCF-KIT (R-HSA-1433557)	3/43	3.59E-02	PTPN6;STAT1;CHEK1
ER-Phagosome pathway (R-HSA-1236974)	4/83	3.61E-02	PSME4;TAP1;HLA-F;HLA-B
SUMOylation of DNA replication proteins (R-HSA-4615885)	3/45	3.93E-02	TOP2A;NUP210;AURKB
Polo-like kinase mediated events (R-HSA-156711)	2/16	4.23E-02	MYBL2;CENPF

Supplementary Table 4. Results of the age-related predicted target gene enrichment analyses

miRNAs	Enrichment of predicted target genes higher expressed with age	
Lower expressed with age	p-value	Genes (bold=significantly negatively correlated with miRNA)
miR-146b-5p	1.13E-02	**RIMS2**, **IGSF1**, **SLITRK3**, **NRP2**, XKR4
miR-142-5p	2.42E-05	**TENM3**, **SH3D19**, **KIT**, CSMD3, SLC24A2, LRP1B, LPHN3, TMOD1, TMEFF2, TMTC1, MPPED2, MPP6, DLG2, WNT2B, SLC23A2, PRKCA
miR-146a-5p	1.13E-02	**RIMS2**, **IGSF1**, SLITRK3, NRP2, XKR4
miR-409-3p	3.19E-02	MME, NR3C2, FGF9, NRP2, GHR
